# Differential impairment of cerebrospinal fluid synaptic biomarkers in the genetic forms of frontotemporal dementia

**DOI:** 10.1186/s13195-022-01042-3

**Published:** 2022-08-31

**Authors:** Aitana Sogorb-Esteve, Johanna Nilsson, Imogen J. Swift, Carolin Heller, Martina Bocchetta, Lucy L. Russell, Georgia Peakman, Rhian S. Convery, John C. van Swieten, Harro Seelaar, Barbara Borroni, Daniela Galimberti, Raquel Sanchez-Valle, Robert Laforce, Fermin Moreno, Matthis Synofzik, Caroline Graff, Mario Masellis, Maria Carmela Tartaglia, James B. Rowe, Rik Vandenberghe, Elizabeth Finger, Fabrizio Tagliavini, Isabel Santana, Chris R. Butler, Simon Ducharme, Alexander Gerhard, Adrian Danek, Johannes Levin, Markus Otto, Sandro Sorbi, Isabelle Le Ber, Florence Pasquier, Johan Gobom, Ann Brinkmalm, Kaj Blennow, Henrik Zetterberg, Jonathan D. Rohrer, on behalf of the GENetic FTD Initiative, Annabel Nelson, Arabella Bouzigues, Caroline V Greaves, David Cash, David L Thomas, Emily Todd, Hanya Benotmane, Jennifer Nicholas, Kiran Samra, Rachelle Shafei, Carolyn Timberlake, Thomas Cope, Timothy Rittman, Alberto Benussi, Enrico Premi, Roberto Gasparotti, Silvana Archetti, Stefano Gazzina, Valentina Cantoni, Andrea Arighi, Chiara Fenoglio, Elio Scarpini, Giorgio Fumagalli, Vittoria Borracci, Giacomina Rossi, Giorgio Giaccone, Giuseppe Di Fede, Paola Caroppo, Pietro Tiraboschi, Sara Prioni, Veronica Redaelli, David Tang-Wai, Ekaterina Rogaeva, Miguel Castelo-Branco, Morris Freedman, Ron Keren, Sandra Black, Sara Mitchell, Christen Shoesmith, Robart Bartha, Rosa Rademakers, Jackie Poos, Janne M. Papma, Lucia Giannini, Rick van Minkelen, Yolande Pijnenburg, Benedetta Nacmias, Camilla Ferrari, Cristina Polito, Gemma Lombardi, Valentina Bessi, Michele Veldsman, Christin Andersson, Hakan Thonberg, Linn Öijerstedt, Vesna Jelic, Paul Thompson, Tobias Langheinrich, Albert Lladó, Anna Antonell, Jaume Olives, Mircea Balasa, Nuria Bargalló, Sergi Borrego-Ecija, Alexandre de Mendonça, Ana Verdelho, Carolina Maruta, Catarina B. Ferreira, Gabriel Miltenberger, Frederico Simões do Couto, Alazne Gabilondo, Ana Gorostidi, Jorge Villanua, Marta Cañada, Mikel Tainta, Miren Zulaica, Myriam Barandiaran, Patricia Alves, Benjamin Bender, Carlo Wilke, Lisa Graf, Annick Vogels, Mathieu Vandenbulcke, Philip Van Damme, Rose Bruffaerts, Koen Poesen, Pedro Rosa-Neto, Serge Gauthier, Agnès Camuzat, Alexis Brice, Anne Bertrand, Aurélie Funkiewiez, Daisy Rinaldi, Dario Saracino, Olivier Colliot, Sabrina Sayah, Catharina Prix, Elisabeth Wlasich, Olivia Wagemann, Sandra Loosli, Sonja Schönecker, Tobias Hoegen, Jolina Lombardi, Sarah Anderl-Straub, Adeline Rollin, Gregory Kuchcinski, Maxime Bertoux, Thibaud Lebouvier, Vincent Deramecourt, Beatriz Santiago, Diana Duro, Maria João Leitão, Maria Rosario Almeida, Miguel Tábuas-Pereira, Sónia Afonso

**Affiliations:** 1 UK Dementia Research Institute at University College London, UCL Queen Square Institute of Neurology, London, UK; 2 Dementia Research Centre, Department of Neurodegenerative Disease, UCL Queen Square Institute of Neurology, London, WC1N 3BG UK; 3 Institute of Neuroscience and Physiology, The Sahlgrenska Academy at the University of Gothenburg, 43180 Mölndal, Sweden; 4 Department of Neurology, Erasmus Medical Centre, Rotterdam, The Netherlands; 5 Centre for Neurodegenerative Disorders, Department of Clinical and Experimental Sciences, University of Brescia, Brescia, Italy; 6 Department of Biomedical, Surgical and Dental Sciences, University of Milan, Milan, Italy; 7 Fondazione IRCCS Ca’ Granda, Ospedale Maggiore Policlinico, Milan, Italy; 8 Alzheimer’s Disease and Other Cognitive Disorders Unit, Neurology Service, Hospital ClínicInstitut d’Investigacións Biomèdiques August Pi I Sunyer, University of Barcelona, Barcelona, Spain; 9 Clinique Interdisciplinaire de MémoireDépartement Des Sciences Neurologiques, CHU de Québec, and Faculté de Médecine, Université Laval, Quebec City, QC Canada; 10 Cognitive Disorders Unit, Department of Neurology, Donostia University Hospital, San Sebastian, Gipuzkoa, Spain; 11 Neuroscience Area, Biodonostia Health Research Institute, San Sebastian, Gipuzkoa, Spain; 12 Department of Neurodegenerative Diseases, Hertie-Institute for Clinical Brain Research and Center of Neurology, University of Tübingen, Tübingen, Germany; 13 Center for Neurodegenerative Diseases (DZNE), Tübingen, Germany; 14 Center for Alzheimer Research, Division of Neurogeriatrics, Department of Neurobiology, Care Sciences and Society, BioclinicumKarolinska Institutet, Solna, Sweden; 15 Unit for Hereditary Dementias, Theme Aging, Karolinska University Hospital, Solna, Sweden; 16 Sunnybrook Health Sciences Centre, Sunnybrook Research Institute, University of Toronto, Toronto, Canada; 17 Tanz Centre for Research in Neurodegenerative Diseases, University of Toronto, Toronto, Canada; 18 Department of Clinical Neurosciences and Cambridge University Hospitals NHS Trust and Medical Research Council Cognition and Brain Sciences Unit, University of Cambridge, Cambridge, UK; 19 Laboratory for Cognitive Neurology, Department of Neurosciences, KU Leuven, Louvain, Belgium; 20 Neurology Service, University Hospitals Leuven, Louvain, Belgium; 21 Leuven Brain Institute, KU Leuven, Louvain, Belgium; 22 Department of Clinical Neurological Sciences, University of Western Ontario, London, ON Canada; 23 Fondazione IRCCS Istituto Neurologico Carlo Besta, Milan, Italy; 24 Faculty of Medicine, University Hospital of Coimbra (HUC), Neurology Service, University of Coimbra, Coimbra, Portugal; 25 Center for Neuroscience and Cell Biology, Faculty of Medicine, University of Coimbra, Coimbra, Portugal; 26 Nuffield Department of Clinical Neurosciences, Medical Sciences Division, University of Oxford, Oxford, UK; 27 Department of Brain Sciences, Imperial College London, London, UK; 28 Department of Psychiatry, Douglas Mental Health University Institute, McGill University, Montreal, Canada; 29 McConnell Brain Imaging Centre, Department of Neurology & Neurosurgery, Montreal Neurological Institute, McGill University, Montreal, Canada; 30 Division of Neuroscience and Experimental Psychology, Wolfson Molecular Imaging Centre, University of Manchester, Manchester, UK; 31 Departments of Geriatric Medicine and Nuclear Medicine, University of Duisburg-Essen, Duisburg, Germany; 32 Neurologische Klinik Und Poliklinik, Ludwig-Maximilians-Universität, Munich, Germany; 33 German Center for Neurodegenerative Diseases (DZNE), Munich, Germany; 34 Munich Cluster of Systems Neurology, Munich, Germany; 35 Department of Neurology, University of Ulm, Ulm, Germany; 36 Department of Neurofarba, University of Florence, Florence, Italy; 37 IRCCS Fondazione Don Carlo Gnocchi, Florence, Italy; 38 Sorbonne Université, Paris Brain Institute – Institut du Cerveau – ICM, Inserm U1127, CNRS UMR 7225, AP-HP - Hôpital Pitié-Salpêtrière, Paris, France; 39 Centre de Référence Des Démences Rares Ou Précoces, IM2A, Département de Neurologie, AP-HP - Hôpital Pitié-Salpêtrière, Paris, France; 40 Département de Neurologie, AP-HP - Hôpital Pitié-Salpêtrière, Paris, France; 41 Reference Network for Rare Neurological Diseases (ERN-RND), Tübingen, Germany; 42 University of Lille, Lille, France; 43 Inserm, 1172, Lille, France; 44 CHU, CNR-MAJ, Labex Distalz, LiCEND, Lille, France; 45 Department of Psychiatry and Neurochemistry, Institute of Neuroscience and Physiology, The Sahlgrenska Academy at the University of Gothenburg, Mölndal, Sweden; 46 Clinical Neurochemistry Laboratory, Sahlgrenska University Hospital, Mölndal, Sweden; 47 Hong Kong Center for Neurodegenerative Diseases, Sha Tin, Hong Kong, China

**Keywords:** Frontotemporal dementia, Synaptic dysfunction, Biomarkers

## Abstract

**Background:**

Approximately a third of frontotemporal dementia (FTD) is genetic with mutations in three genes accounting for most of the inheritance: *C9orf72*,* GRN*, and *MAPT*. Impaired synaptic health is a common mechanism in all three genetic variants, so developing fluid biomarkers of this process could be useful as a readout of cellular dysfunction within therapeutic trials.

**Methods:**

A total of 193 cerebrospinal fluid (CSF) samples from the GENetic FTD Initiative including 77 presymptomatic (31 *C9orf72*, 23 *GRN*, 23 *MAPT*) and 55 symptomatic (26 *C9orf72*, 17 *GRN*, 12 *MAPT*) mutation carriers as well as 61 mutation-negative controls were measured using a microflow LC PRM-MS set-up targeting 15 synaptic proteins: AP-2 complex subunit beta, complexin-2, beta-synuclein, gamma-synuclein, 14–3-3 proteins (eta, epsilon, zeta/delta), neurogranin, Rab GDP dissociation inhibitor alpha (Rab GDI alpha), syntaxin-1B, syntaxin-7, phosphatidylethanolamine-binding protein 1 (PEBP-1), neuronal pentraxin receptor (NPTXR), neuronal pentraxin 1 (NPTX1), and neuronal pentraxin 2 (NPTX2). Mutation carrier groups were compared to each other and to controls using a bootstrapped linear regression model, adjusting for age and sex.

**Results:**

CSF levels of eight proteins were increased only in symptomatic *MAPT* mutation carriers (compared with controls) and not in symptomatic *C9orf72* or *GRN* mutation carriers: beta-synuclein, gamma-synuclein, 14–3-3-eta, neurogranin, Rab GDI alpha, syntaxin-1B, syntaxin-7, and PEBP-1, with three other proteins increased in *MAPT* mutation carriers compared with the other genetic groups (AP-2 complex subunit beta, complexin-2, and 14–3-3 zeta/delta). In contrast, CSF NPTX1 and NPTX2 levels were affected in all three genetic groups (decreased compared with controls), with NPTXR concentrations being affected in *C9orf72* and *GRN* mutation carriers only (decreased compared with controls). No changes were seen in the CSF levels of these proteins in presymptomatic mutation carriers. Concentrations of the neuronal pentraxins were correlated with brain volumes in the presymptomatic period for the *C9orf72* and *GRN* groups, suggesting that they become abnormal in proximity to symptom onset.

**Conclusions:**

Differential synaptic impairment is seen in the genetic forms of FTD, with abnormalities in multiple measures in those with *MAPT* mutations, but only changes in neuronal pentraxins within the *GRN* and *C9orf72* mutation groups. Such markers may be useful in future trials as measures of synaptic dysfunction, but further work is needed to understand how these markers change throughout the course of the disease.

**Supplementary Information:**

The online version contains supplementary material available at 10.1186/s13195-022-01042-3.

## Background

Frontotemporal dementia (FTD) is the most common cause of dementia affecting people under the age of 60. Clinically, it presents heterogeneously, manifesting as a behavioural variant (bvFTD), language impairment (primary progressive aphasia, PPA), or with a motor presentation (either amyotrophic lateral sclerosis, FTD-ALS, or an atypical parkinsonian disorder). The FTD spectrum is characteristically associated with neuronal dysfunction and loss in the frontal and temporal lobes, but more widespread cortical, subcortical, cerebellar, and brainstem involvement is now also recognized [1]. Around a third of people with FTD have a genetic cause, with the most common mutations occurring in three genes: *GRN* (progranulin), *C9orf72* (chromosome 9 open reading frame 72), and *MAPT* (microtubule-associated protein tau) [2, 3]. Lastly, the underlying pathology of FTD can be one of three forms: cellular inclusions containing abnormal forms of tau, TAR DNA-binding protein 43 (TDP-43), or FET proteins (*fused in sarcoma* (FUS), *Ewing sarcoma* (EWS), and *TATA-binding associated factor 15* (TAF15)) [4]*.*

The interaction between clinical phenotype, neuroanatomical features, genotype, and pathology is complex and means that FTD can be hard to diagnose (particularly its specific pathological form during life) and difficult to track over time. To further examine some of these outstanding issues in the FTD field, researchers have aimed to develop fluid biomarkers, measured typically in the cerebrospinal fluid (CSF), serum, or plasma using a variety of different techniques. Biomarkers can provide an insight into the underlying pathophysiology of FTD and in the context of clinical trials could offer a direct experimental medicine approach to understanding the molecular mechanisms through measurement of biofluids pre- and post-intervention [5].

Whilst some pathways are specific to certain pathogenetic forms of FTD, studies in recent years have particularly highlighted the importance of synaptic health [6–10] as one of the major pathophysiological mechanisms across the FTD spectrum. Progressive synaptic dysfunction and loss have been shown to occur in FTD, raising the hypothesis that any changes in synaptic proteins in brain tissue may also be reflected in their concentrations within the CSF (and potentially the blood) of people with FTD. In this study, we investigated a panel of CSF synaptic markers in presymptomatic and symptomatic people with genetic FTD from the GENetic Frontotemporal dementia Initiative (GENFI), hypothesizing that we would find differential abnormalities across *MAPT*, *GRN*, and *C9orf72* mutation carriers.

## Methods

### Participants and sample collection

Participants were recruited from the GENFI study, which follows patients with FTD due to a pathogenic mutation in *MAPT*,* GRN*, or *C9orf72* (symptomatic mutation carriers) and healthy at-risk first-degree relatives (either presymptomatic mutation carriers or non-carriers) [11]. We included 77 presymptomatic mutation carriers (31 *C9orf72*, 23 *GRN*, 23 *MAPT*), 55 symptomatic mutation carriers (26 *C9orf72*, 17 *GRN*, 12 *MAPT*), and 61 non-carriers. Age at the time of CSF sample collection was not statistically different within each group, and a similar percentage of males and females was included (Table 1). Participants were assessed using a standardized history and examination and classified as symptomatic if they met consensus diagnostic criteria [12, 13]. The CDR Dementia Staging Instrument with National Alzheimer Coordinating Centre Frontotemporal Lobar Degeneration component (CDR® plus NACC FTLD) was used to assess disease severity. Local ethics committees at each site approved the study, and all participants provided written informed consent.

**Table 1:** Demographics of participants in the study. *N* number of participants. Values are shown as mean (standard deviation)

	**Non-carriers**	**Presymptomatic carriers**			**Symptomatic carriers**		
		***C9orf72***	***GRN***	***MAPT***	***C9orf72***	***GRN***	***MAPT***
***N***	61	31	23	23	26	17	12
**Age at CSF sampling**	44.9 (13.2)	42.2 (11.1)	45.3 (13.8)	40.3 (10.6)	63.3 (9.2)	63.1 (7.5)	61.4 (7.4)
**Sex (% females)**	62.3	51.6	47.8	65.2	38.5	52.9	50
**CDR plus NACC FTLD sum of boxes**	0.2 (0.5)	0.3 (0.6)	0.3 (1)	0.6 (0.7)	10.5 (5.6)	9.4 (5.4)	8.3 (4.5)
**Plasma NfL (pg/mL)**	8.7 (6.3)	10.6 (11.8)	7.5 (3.5)	7.2 (3.3)	49.1 (29.2)	65.1 (54.6)	19.8 (2.7)
**Total brain volume (as a percentage of total intracranial volume)**	80.8 (3.3)	79.7 (2.6)	80.6 (3.1)	80.9 (2.2)	72.7 (3.5)	72.6 (4.2)	74.5 (3.5)

### CSF collection and LC–MS/MS analysis

**Fig. 1:**
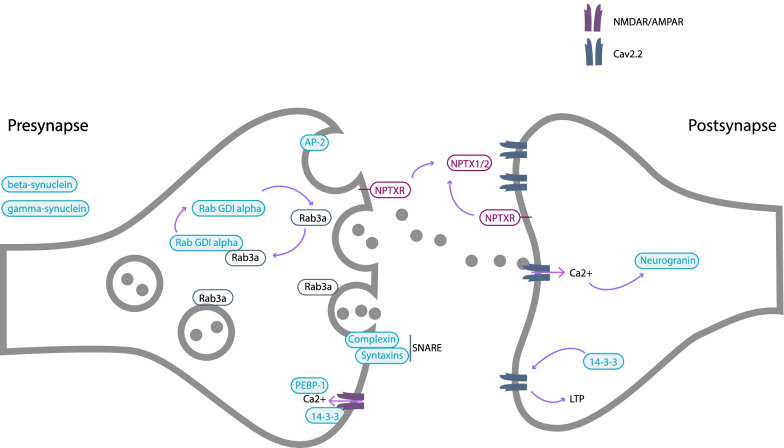
Diagrammatic representation of the synapse and the role of the different synaptic proteins included within the mass spectrometry panel. Adapted from Nilsson et al. [14]

**Fig. 2:**
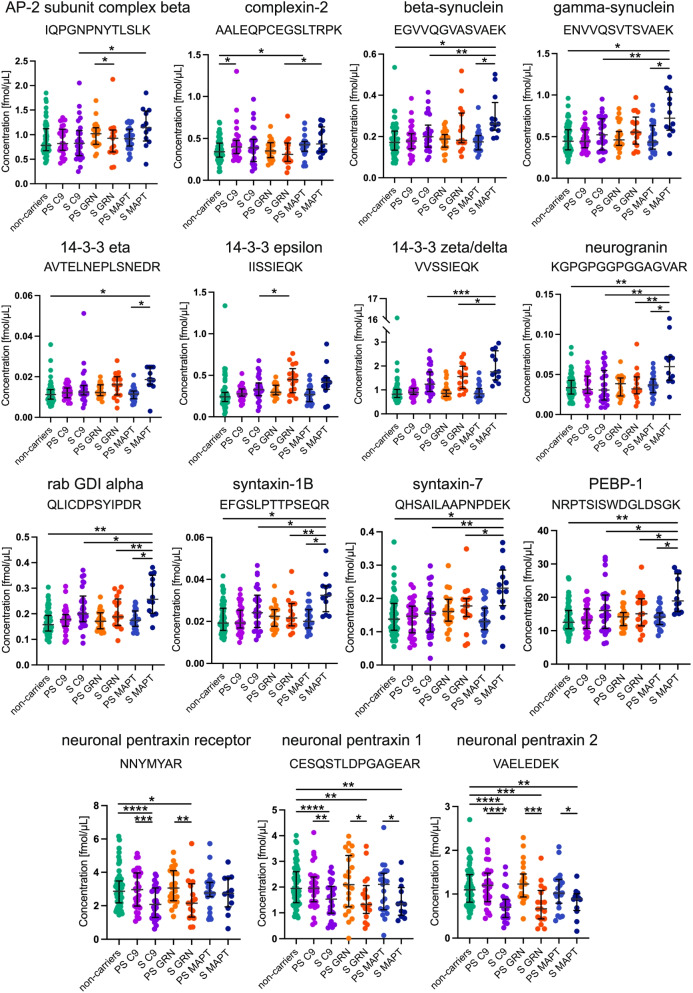
Cerebrospinal fluid (CSF) concentrations of the synaptic panel proteins in the GENFI cohort including 23 presymptomatic *MAPT* (PS MAPT), 31 *C9orf72* (PS C9), and 23 *GRN* (PS GRN) mutation carriers and 12 symptomatic *MAPT* (S MAPT), 26 *C9orf72* (S C9), and 17 *GRN* (S GRN) mutation carriers and 61 non-carriers. The results are shown in fmol/μL. *p*-values: *p ≤ 0.05, **p ≤ 0.01, ***p ≤ 0.001, and ****p ≤ 0.0001. The bars indicate the median and the IQR. Only one peptide per protein is shown as discussed in the “Methods” section. Specific means, IC, and *p*-values are shown in Additional file 1: Table S2

CSF was collected in polypropylene tubes through a lumbar puncture and centrifuged to remove insoluble material and cells. Supernatants were aliquoted and stored at − 80 °C within 2 h after withdrawal. For the mass spectrometry analysis, sample preparation was performed as described previously [14]. Briefly, to 100 µL of CSF, a mixture of stable isotope-labeled peptides (internal standard) was added (25 µL, 0.032 pmol/µL, JPT Peptide Technologies, Berlin, Germany; SpikeTides L). This was then followed by a stepwise protocol of reduction, alkylation, and tryptic digestion and lastly solid-phase extraction for purification purposes (for detailed sample preparation, refer to Additional file 3: Appendix 2). LC–MS/MS analysis was performed using a microflow HPLC, equipped with a Hypersil Gold reversed-phase column (100 × 2.1 mm, particle size 1.9 µm, Thermo Fisher Scientific), and a Triple Quadrupole Mass Spectrometer (6495 Triple Quadrupole LC/MS system, Agilent Technologies). LC–MS settings are shown in Additional file 3: Appendix 2. To monitor the performance of the assay over time, quality control (QC) sample replicates were injected at regular intervals during runs. The panel of synaptic markers included (Fig. 1) AP-2 complex subunit beta, complexin-2, beta-synuclein, gamma-synuclein, 14–3-3 proteins (eta, epsilon, zeta/delta), neurogranin, Rab GDP dissociation inhibitor alpha (Rab GDI alpha), syntaxin-1B, syntaxin-7, phosphatidylethanolamine-binding protein 1 (PEBP-1), neuronal pentraxin receptor (NPTXR), neuronal pentraxin 1 (NPTX1), and neuronal pentraxin 2 (NPTX2). Table 2 shows the proteins and their respective proteotypic peptides targeted in the multiple reaction monitoring mass spectrometry analysis [14] as well as their analytical performance. For the proteins for which more than one peptide was quantified, the peptide with the best analytical performance (lowest coefficient of variation) is discussed in the main manuscript and shown in Fig. 2.

**Table 2:** The 15 synaptic proteins and their respective peptides included in the panel. In the far right-hand column, the repeatability (presented as coefficients of variation (CV)) for proteins/peptides quantified in the study is shown. Proteins/peptides marked as bold were included in the statistical analysis

Protein	Accession	Sequence	Position	Repeatability (CV%)
**AP-2 complex subunit beta**	P63010	NVEGQDMLYQSLK	[880–892]	11.1
		**IQPGNPNYTLSLK**	[905–917]	7.1
**Complexin-2**	Q6PUV4	**AALEQPCEGSLTRPK**	[84–98]	15.6
**Beta-synuclein**	Q16143	**EGVVQGVASVAEK**	[46–58]	12.3
**Gamma-synuclein**	O76070	**ENVVQSVTSVAEK**	[46–58]	8.3
**14–3-3 protein eta**	Q04917	**AVTELNEPLSNEDR**	[29–42]	14.7
**14–3-3 protein epsilon**	P62258	**IISSIEQK**	[62–69]	21.7
**14–3-3 protein zeta/delta**	P63104	**VVSSIEQK**	[61–68]	6.7
**Neurogranin**	Q92686	**KGPGPGGPGGAGVAR**	[54–68]	15.0
**Rab GDI alpha**	P31150	**QLICDPSYIPDR**	[279–290]	11.7
**Syntaxin-1B**	P61266	**QHSAILAAPNPDEK**	[56–69]	16.5
**Syntaxin-7**	O15400	**EFGSLPTTPSEQR**	[72–84]	9.3
**PEBP-1**	P30086	LYEQLSGK	[180–187]	13.5
		**NRPTSISWDGLDSGK**	[48–62]	10.6
**Neuronal pentraxin receptor**	O95502	**NNYMYAR**	[302–308]	5.7
		LVEAFGGATK	[479–488]	9.5
**Neuronal pentraxin-1**	Q15818	LENLEQYSR	[144–152]	16.9
		**CESQSTLDPGAGEAR**	[89–103]	6.1
**Neuronal pentraxin-2**	P47972	**VAELEDEK**	[177–184]	4.5
		WPVETCEER	[419–428]	8.9

### Other biomarkers

Participants underwent volumetric T1-weighted magnetic resonance imaging according to the harmonized GENFI protocol on a 3T scanner. All images underwent a quality control check, and scans with movement or artefacts were removed from the analysis. Only scans from mutation carriers were included in the correlative analysis: of the 132 participants, 111 scans were available for the analysis: 49 *C9orf72*, 34 *GRN*, and 28 *MAPT* mutation carriers. Neuroanatomical regions of interest were generated as previously described using an automated atlas segmentation propagation and label fusion strategy called geodesic information flow [11]. Specifically, total brain volume and volumes of the frontal, temporal, and parietal cortices were calculated and expressed as a percentage of total intracranial volume (TIV), computed with SPM12 (Statistical Parametric Mapping, Welcome Trust Centre for Neuroimaging, London, UK) running under Matlab R2014b.

Participants also had plasma samples collected as part of the GENFI protocol. Plasma was collected, processed, and stored in aliquots at − 80 °C according to standardized procedures. Plasma neurofilament light chain (NfL) levels were correlated with synaptic proteins with only measures from mutation carriers included: of 132 participants, 108 plasma NfL values were available for the analysis: 47 *C9orf72*, 34 *GRN*, and 27 *MAPT* mutation carriers. Plasma NfL concentration was measured with single molecule array (Simoa) technology using the Neurology 4-Plex A kit (Quanterix, Billerica, USA) on an HD-X Analyzer following the manufacturer’s instructions (Quanterix, Billerica, USA). Measurements were completed in duplicate (all CVs below 15%) over a total of 3 batches, each with an 8-point calibration curve tested in triplicate and 2 controls tested in duplicate, as reported before [15].

### Data processing and statistical analysis

Mass spectrometer data processing was performed in Skyline 20.1 (MacCoss Lab Software). All peaks were visually inspected and adjusted if required for optimal peak area calculation. The relative peptide concentration (fmol/µL) was obtained by the ratio of the total area for each peptide against the total area of the corresponding internal standard (IS) multiplied by the amount of IS added per volume of CSF.

All statistical analyses were performed in STATA (v.16) and RStudio (R version 4.0.2). The Shapiro–Wilk test was performed to determine the normality of distribution of each synaptic marker in each group. The levels of each synaptic protein were compared between the groups using a linear regression model adjusting for age at CSF sample collection and sex; bootstrapping with 2000 repetitions was used if the synaptic measures were not normally distributed.

Spearman correlation coefficients were assessed for the synaptic markers between their values and other biomarker data including normalized volumes of total brain, frontal cortex, temporal cortex, and parietal cortex; plasma NfL; and the CDR plus NACC FTLD sum of boxes score.

## Results

### Concentrations of synaptic markers by genotype

Significant increases in CSF levels of several synaptic proteins were seen in symptomatic *MAPT* mutation carriers compared with controls (Fig. 2, Additional file 1: Table S1): beta-synuclein, gamma-synuclein, 14–3-3 eta, neurogranin, Rab GDI alpha, syntaxin 1B, syntaxin-7, and PEBP-1. CSF levels of all of these proteins except 14–3-3 eta were increased in the symptomatic *MAPT* group compared with the symptomatic *C9orf72* group (as was AP-2 complex subunit beta). Similarly, levels of all of these proteins except 14–3-3 eta and both beta and gamma-synuclein were increased in the symptomatic *MAPT* group compared with the symptomatic *GRN* group (as was complexin-2). Furthermore, 14–3-3 zeta/delta was additionally increased in the symptomatic *MAPT* group compared with both *C9orf72* and *GRN* symptomatic mutation carriers. CSF levels of all of these proteins except syntaxin-7 were increased in the symptomatic *MAPT* mutation carriers compared with the presymptomatic *MAPT* mutation carriers (Fig. 2).

In contrast, CSF concentrations of the neuronal pentraxins were found to be decreased in most of the mutation carrier groups compared with controls. At least one of the peptides measured for NPTXR, NPTX1, and NPTX2 were decreased in symptomatic *C9orf72* and *GRN* mutation carriers compared to controls and to their respective presymptomatic group. One NPTX1 and one NPTX2 peptide were also decreased in the symptomatic *MAPT* group compared to controls and the presymptomatic *MAPT* mutation carriers (Fig. 2).

### Correlations of synaptic markers with other biomarkers

For the synaptic markers that had increased CSF concentrations in *MAPT* mutation carriers, no significant correlations were seen with brain volumes, NfL, or CDR plus NACC FTLD.

However, a number of significant correlations were seen with the CSF levels of neuronal pentraxins across the genetic groups (Table 3). In presymptomatic *C9orf72* mutation carriers, there were significant positive correlations of total brain volume with NPTXR and NPTX2 (*r* = 0.42 and 0.38, respectively). Additionally, there were significant positive correlations in this group with temporal cortex volume for NPTXR (*r* = 0.50, *p* = 0.006) and NPTX2 (0.49, 0.007) and with parietal cortex volume for NPTX1 (0.41, 0.029). Two of the neuronal pentraxins (NPTXR and NPTX2) were significantly negatively correlated with CDR plus NACC FTLD. In the presymptomatic *GRN* group, there were significant positive correlations with the frontal lobe (*r* = 0.52 to 0.53) and parietal lobe (*r* = 0.45 to 0.59) for almost all of the measures. There were no correlations with any of the imaging measures in the presymptomatic *MAPT* group, but there was a significant negative correlation with NfL for NPTX1 (*r* =  − 0.46, *p* = 0.040).

**Table 3:** Correlations of the neuronal pentraxins with brain volumes derived from structural T1-weighted MR imaging (total brain as well as frontal, temporal, and parietal cortical volumes), NfL (plasma neurofilament light chain concentration), and CDR plus NACC FTLD sum of boxes score

		**Presymptomatic *C9orf72***		**Symptomatic *C9orf72***		**Presymptomatic** ***GRN***		**Symptomatic *GRN***		**Presymptomatic *MAPT***		**Symptomatic *MAPT***	
		**Rho**	***p*-value**	**Rho**	***p*-value**	**Rho**	***p*-value**	**Rho**	***p*-value**	**Rho**	***p*-value**	**Rho**	***p*-value**
**NPTXR NNYMYAR**	**Total brain**	**0.42**	**0.025**	0.23	0.365	0.14	0.532	0.48	0.161	0.24	0.303	0.66	0.157
	**Frontal**	0.19	0.321	0.41	0.090	**0.53**	**0.011**	**0.73**	**0.017**	0.15	0.522	0.81	0.053
	**Temporal**	**0.50**	**0.006**	0.14	0.588	0.30	0.182	**0.77**	**0.009**	− 0.06	0.810	0.30	0.564
	**Parietal**	0.11	0.571	0.10	0.704	**0.52**	**0.014**	0.51	0.131	0.17	0.483	0.64	0.170
	**NfL**	0.00	0.994	** − 0.68**	**0.005**	0.02	0.925	0.08	0.821	− 0.14	0.572	0.94	0.016
	**CDR plus NACC FTLD**	** − 0.38**	**0.044**	− 0.29	0.248	0.00	0.997	− 0.28	0.425	0.19	0.422	− 0.21	0.697
**NPTX1 CESQSTLDPGAGEAR**	**Total brain**	0.36	0.052	0.11	0.654	0.34	0.118	0.17	0.633	0.23	0.327	0.40	0.427
	**Frontal lobe**	0.25	0.183	0.06	0.812	**0.52**	**0.012**	0.42	0.225	0.00	0.996	0.54	0.264
	**Temporal lobe**	0.20	0.300	− 0.05	0.833	0.41	0.058	0.40	0.255	− 0.09	0.711	0.01	0.984
	**Parietal lobe**	**0.41**	**0.029**	− 0.24	0.345	**0.59**	**0.004**	0.44	0.205	0.08	0.740	0.50	0.315
	**NfL**	0.04	0.856	− 0.51	0.051	0.07	0.757	− 0.22	0.539	** − 0.46**	**0.040**	0.54	0.348
	**CDR plus NACC FTLD**	0.10	0.601	− 0.07	0.770	− 0.12	0.593	0.06	0.870	− 0.01	0.961	− 0.45	0.373
**NPTX2 VAELEDEK**	**Total brain**	**0.38**	**0.045**	0.38	0.120	0.25	0.257	0.50	0.145	0.31	0.189	**0.95**	**0.004**
	**Frontal lobe**	0.21	0.277	**0.60**	**0.008**	0.41	0.059	**0.80**	**0.006**	0.16	0.489	0.80	0.054
	**Temporal lobe**	**0.49**	**0.007**	0.42	0.080	0.20	0.377	**0.65**	**0.043**	0.12	0.623	0.75	0.085
	**Parietal lobe**	0.18	0.343	0.27	0.274	**0.45**	**0.035**	0.50	0.141	0.11	0.657	0.67	0.148
	**NfL**	0.05	0.786	− 0.48	0.073	0.06	0.781	− 0.08	0.837	− 0.03	0.886	0.67	0.215
	**CDR plus NACC FTLD**	** − 0.37**	**0.048**	** − 0.49**	**0.041**	0.00	0.989	− 0.24	0.497	0.18	0.436	− 0.60	0.204

In the symptomatic *C9orf72* group, NPTXR was significantly negatively correlated with NfL concentration (*r* =  − 0.68), whilst in the symptomatic *GRN* group, NPTXR and NPTX2 positively correlated with both frontal (*r* = 0.73 and 0.80, respectively) and temporal (*r* = 0.77 and 0.65, respectively) lobe volumes. In the symptomatic *MAPT* mutation carriers, there was a significant positive correlation of total brain volume with NPTX2 (*r* = 0.95, *p* = 0.004).

## Discussion

In this study, we showed an increased CSF concentration of multiple synaptic markers in symptomatic *MAPT* mutation carriers. In contrast, concentrations of the neuronal pentraxins were decreased in all three symptomatic genetic groups. Although no group-wise differences in CSF levels were seen presymptomatically, correlations with brain volumes in the *C9orf72* and *GRN* groups suggest that the neuronal pentraxins change in the lead up to symptom onset as the brain volume starts to decrease.

For the proteins found to have abnormal CSF levels in *MAPT* mutations, little is known previously about their involvement in the pathophysiology of FTD. Beta- and gamma-synucleins are present in the proteinaceous aggregates characteristic of the alpha-synucleinopathies [16] although their normal function is still unclear. Previous studies have shown an increase in these markers in the CSF of people with Alzheimer’s disease (AD) [14, 17, 18], but in one prior study of beta-synuclein in the CSF of people with undifferentiated FTD, the levels were normal [17]. The results in this study therefore represent a novel association with *MAPT* mutations.

14–3-3 proteins are highly expressed in the brain, particularly enriched in the presynaptic site and are implicated in synaptic plasticity by acting as modulators of neurotransmission [19]. Although they are established biomarkers for Creutzfeldt-Jakob disease, they have also been genetically linked to AD and found to colocalize with tau in the neurofibrillary tangles as well as in Lewy bodies in Parkinson’s disease [20]. Furthermore, increased levels of 14–3-3 protein have previously been reported in CSF from people with FTD (not differentiated into a specific form) in a single study, as well as in people with AD [14, 21]. Here, we show increased levels of 14–3-3 eta protein in the symptomatic *MAPT* mutation carriers when compared with the non-carrier group, and for 14–3-3 zeta/delta, when compared with the other symptomatic groups. These results could potentially indicate a specific relationship with tau pathology, related to the deposition in neurofibrillary tangles of 14–3-3 proteins. However, there are also trends to an increase in some of the 14–3-3 proteins in *GRN* mutations, so genotype differences may not be as clear here.

Neurogranin has been well-studied in the AD field as a fluid biomarker over recent years [22, 23]. It is a postsynaptic molecule involved in long-term potentiation and synaptic plasticity mediated by Ca^2+^ and calmodulin signalling pathways [24, 25]. In CSF, neurogranin shows an increase in people with AD compared with controls [14, 26–28]. Furthermore, increased concentrations of neurogranin in CSF predict cognitive decline from mild cognitive impairment (MCI) to AD [29, 30]. In a previous study, FTD levels of neurogranin were not significantly different to controls [31], although when stratified into those with tau and TDP-43 pathology, there was a trend for an increase in the tau group. A further study has also shown that neurogranin was significantly decreased in comparison with controls in plasma exosomes from people with FTD [32]. In this study, we show an increase in neurogranin levels in symptomatic *MAPT* mutation carriers, again suggesting a specific relationship with tau pathology.

AP-2 complex subunit beta, the syntaxins, Rab GDI alpha, and PEBP-1 are all implicated in the process of synapse vesicle exocytosis and neurotransmitter release at the synaptic cleft, and their CSF levels have previously been shown to be abnormal in AD [14, 33–36]. Syntaxins participate in the formation of the soluble *N*-ethylmaleimide-sensitive factor attachment receptor (SNARE) complex, where they participate in synapse vesicle exocytosis together with complexin-2, which modulates the function of the SNARE complex [33, 37]. The levels of syntaxin 1B have been shown to be increased at an early preclinical stage in the CSF of people likely to develop AD, even before core CSF biomarkers for neurodegeneration [34]. None of these proteins has been previously studied in FTD, but given their increase also in AD, it may be that these are all tau-specific markers of synaptic dysfunction, and further study in other primary tauopathies would be important.

Finally, we showed changes across all three genetic groups in the neuronal pentraxins. Pentraxins are multifunctional proteins divided into different groups according to their length. They are not exclusively localized in the central nervous system (CNS) and are involved in the inflammatory response as well as synaptic plasticity among other functions [35]. The sub-family of neuronal pentraxins includes the soluble neuronal pentraxins 1 (NPTX1) and 2 (NPTX2) and the transmembrane neuronal pentraxin receptor (NPTXR). NPTXs are implicated in synaptic plasticity, synapse formation, and remodelling [36]. Both the two secreted NPTX1 and NPTX2 and the transmembrane receptor NPTXR have been found in several studies to be decreased in CSF in AD compared with controls [14, 38–41] and appear to be markers of disease progression in AD [42–44]. Recent proteomic studies have shown decreased levels of NPTXR in symptomatic genetic FTD, in all three genetic groups [45], and in sporadic bvFTD and PPA [46]. Two further studies additionally showed that NPTX2 was decreased in symptomatic mutation carriers in all three groups compared with controls using antibody-based approaches [47, 48], and one of these studies showed that NPTX1 was decreased in *C9orf72* and *MAPT* mutation carriers [48]. In our study, NPTX1 and NPTX2 were significantly decreased in all symptomatic groups, but NPTXR was only decreased in *C9orf72* and *GRN* mutation carriers. In one prior study, levels of NPTX2 in CSF correlated with disease progression, with the suggestion also that NPTX2 levels change just prior to symptom onset [48, 49]. The correlations of the neuronal pentraxins with brain volumes in the presymptomatic *C9orf72* and *GRN* mutation carriers suggest that for these two groups, NPTXR, NPTX1, and NPTX2 change in proximity to symptom onset as brain volumes start to decrease. In the symptomatic *C9orf72* expansion carriers, the neuronal pentraxin levels correlated with NfL, which can be variable in concentration in this group [50]. However, it suggests that at least in *C9orf72* expansion carriers, the neuronal pentraxins may be a measure of disease intensity and speed of progression. In contrast, in the *GRN* and *MAPT* mutation carriers, neuronal pentraxin concentrations correlated with brain volumes, suggesting that here they may be a measure of disease severity rather than intensity.

### Limitations

Limitations of the study include the limited number of CSF samples in each group after stratification. However, this is the largest study so far of synaptic biomarkers in this uncommon disease and replicates prior work on neuronal pentraxins. Further work to replicate the findings in other *MAPT* mutation cohorts (and other primary tauopathies) as well as longitudinal analysis within the GENFI cohort will be important. Lastly, the specific synaptic markers panel used here was designed specifically to target AD pathology based on prior research and selected from a broad proteomic study in an AD cohort [51]. It may therefore be that this panel was more likely to find abnormalities in tauopathies and that other synaptic proteins not included in the panel might be better biomarkers for assessing synaptic dysfunction in FTD, particularly in those with TDP-43 pathology.

## Conclusion

In this study, we show differential involvement of synaptic proteins in the three main genetic groups accounting for familial FTD. Our results suggest that different pathways may be related to synaptic health in relation to the underlying proteinopathy found in each mutation. Future studies will focus on replication of these findings, longitudinal analyses of these measures, and a broader proteomic study to better customise a synaptic biomarker panel targeted to different forms of FTD.

## Supplementary Information

**Additional file 1:** Supplementary tables.

**Additional file 2: Appendix 1.** List of GENFI consortium authors.

**Additional file 3: Appendix 2.** IS preparation and LC-MS settings for theanalysis of the synaptic protein panel.

## Data Availability

The datasets used and/or analysed during the current study are available from the corresponding author on reasonable request.

## Abbreviations

FTD: Frontotemporal dementia
GENFI: GENetic Frontrotemporal dementia Initiative
bvFTD: Behavioural variant FTD
PPA: Primary progressive aphasias
FTD-ALS: FTD with amyotrophic lateral sclerosis
*GRN*: Progranulin gene
*C9orf72*: Chromosome 9 open reading frame 72 gene
*MAPT*: Microtubule-associated protein tau gene
TDP-43: TAR DNA-binding protein 43
FET: FUS, EWS, and TAF15 proteins
FUS: Fused in sarcoma
EWS: Ewing sarcoma
TAF15: TATA-binding associated factor 15
CSF: Cerebrospinal fluid
CDR® plus NACC FTLD: CDR Dementia Staging Instrument with National Alzheimer Coordinating Centre Frontotemporal Lobar Degeneration Component
LC–MS/MS: Liquid chromatography-mass spectrometry/mass spectrometry
HPLC: High-pressure liquid chromatography
Rab GDI alpha: Rab GDP dissociation inhibitor alpha
PEBP-1: Phosphatidylethanolamine-binding protein 1
NPTXR: Neuronal pentraxin receptor
NPTX1: Neuronal pentraxin 1
NPTX2: Neuronal pentraxin 2
SPM12: Statistical Parametric Mapping
NfL: Neurofilament light chain
IS: Internal standard
AD: Alzheimer’s disease
MCI: Mild cognitive impairment
SNARE: Soluble *N*-ethylmaleimide-sensitive factor attachment receptor
CNS: Central nervous system
