# Supplementary material for: Differential impairment of cerebrospinal fluid synaptic biomarkers in the genetic forms of frontotemporal dementia
Source: Alzheimers Res Ther. 2022 Aug 31;14:118. doi: 10.1186/s13195-022-01042-3 (PMC9429339; doi:10.1186/s13195-022-01042-3)
Supplement: Supplementary file 1 — Additional file 1: Supplementary tables. [file 13195_2022_1042_MOESM1_ESM.docx]

**Supplementary Table 1 a) to t). Adjusted mean differences in the 20 synaptic panel peptide concentrations between groups with observed contrast, 95% bootstrapped confidence intervals p-values (significant in bold).**

**a) AP-2 subunit complex beta (IQPGNPNYTLSLK)**

|  |  | **Controls** | ***C9orf72*** | | | | | ***GRN*** | | | | ***MAPT*** | | | |
| --- | --- | --- | --- | --- | --- | --- | --- | --- | --- | --- | --- | --- | --- | --- | --- |
|  |  |  | **PS** | | | **S** | | **PS** | | **S** | | **PS** | | **S** | |
| **controls** | |  | 0.005 | | | -0.148 | | 0.108 | | -0.133 | | 0.061 | | 0.135 | |
|  |  |  | -0.119 | | 0.128 | -0.317 | 0.022 | -0.024 | 0.241 | -0.346 | 0.080 | -0.061 | 0.183 | -0.112 | 0.382 |
|  |  |  | 0.942 | | | 0.088 | | 0.108 | | 0.221 | | 0.325 | | 0.283 | |
| ***C9orf72*** | **PS** |  |  |  |  | -0.152 | | 0.104 | | -0.138 | | 0.057 | | 0.131 | |
|  |  |  |  |  |  | -0.336 | 0.031 | -0.042 | 0.249 | -0.360 | 0.085 | -0.079 | 0.193 | -0.123 | 0.384 |
|  |  |  |  |  |  | 0.104 | | 0.162 | | 0.226 | | 0.415 | | 0.312 | |
|  | **S** |  |  |  |  |  |  | 0.256 | | 0.015 | | 0.209 | | 0.283 | |
|  |  |  |  |  |  |  |  | 0.071 | 0.441 | -0.229 | 0.258 | 0.022 | 0.396 | 0.018 | 0.547 |
|  |  |  |  |  |  |  |  | **0.007** | | 0.906 | | **0.028** | | **0.036** | |
| ***GRN*** | **PS** |  |  |  |  |  |  |  |  | -0.242 | | -0.047 | | 0.027 | |
|  |  |  |  |  |  |  |  |  |  | -0.467 | -0.016 | -0.190 | 0.096 | -0.229 | 0.283 |
|  |  |  |  |  |  |  |  |  |  | **0.036** | | 0.517 | | 0.838 | |
|  | **S** |  |  |  |  |  |  |  |  |  |  | 0.194 | | 0.268 | |
|  |  |  |  |  |  |  |  |  |  |  |  | -0.033 | 0.422 | -0.029 | 0.565 |
|  |  |  |  |  |  |  |  |  |  |  |  | 0.094 | | 0.077 | |
| ***MAPT*** | **PS** |  |  |  |  |  |  |  |  |  |  |  |  | 0.074 | |
|  |  |  |  |  |  |  |  |  |  |  |  |  |  | -0.187 | -0.017 |
|  |  |  |  |  |  |  |  |  |  |  |  |  |  | 0.579 | |
|  | **S** |  |  |  |  |  |  |  |  |  |  |  |  |  |  |
|  |  |  |  |  |  |  |  |  |  |  |  |  |  |  |  |
|  |  |  |  |  |  |  |  |  |  |  |  |  |  |  |  |

**b) AP-2 subunit complex beta (NVEGQDMLYQSLK)**

|  |  | **Controls** | | ***C9orf72*** | | | | ***GRN*** | | | | ***MAPT*** | | | |
| --- | --- | --- | --- | --- | --- | --- | --- | --- | --- | --- | --- | --- | --- | --- | --- |
|  |  |  |  | **PS** | | **S** | | **PS** | | **S** | | **PS** | | **S** | |
| **controls** | |  | | 0.005 | | -0.206 | | 0.196 | | -0.194 | | 0.219 | | 0.733 | |
|  |  |  |  | -0.336 | 0.347 | -0.719 | 0.307 | -0.213 | 0.604 | -0.808 | 0.421 | -0.162 | 0.599 | -0.187 | 1.653 |
|  |  |  |  | 0.976 | | 0.431 | | 0.348 | | 0.536 | | 0.260 | | 0.118 | |
| **C9orf72** | **PS** |  |  |  |  | -0.212 | | 0.190 | | -0.199 | | 0.213 | | 0.728 |  |
|  |  |  |  |  |  | -0.745 | 0.322 | -0.232 | 0.613 | -0.831 | 0.433 | -0.178 | 0.604 | -0.201 | 1.657 |
|  |  |  |  |  |  | 0.437 | | 0.377 | | 0.537 | | 0.285 | | 0.125 | |
|  | **S** |  |  |  |  |  |  | 0.402 | | 0.013 | | 0.425 | | 0.939 | |
|  |  |  |  |  |  |  |  | -0.156 | 0.960 | -0.671 | 0.696 | -0.146 | 0.995 | -0.033 | 1.912 |
|  |  |  |  |  |  |  |  | 0.158 | | 0.971 | | 0.144 | | 0.058 | |
| ***GRN*** | **PS** |  |  |  |  |  |  |  |  | -0.389 | | 0.023 | | 0.537 | |
|  |  |  |  |  |  |  |  |  |  | -1.044 | 0.265 | -0.430 | 0.476 | -0.412 | 1.487 |
|  |  |  |  |  |  |  |  |  |  | 0.244 | | 0.921 | | 0.267 | |
|  | **S** |  |  |  |  |  |  |  |  |  |  | 0.412 | | 0.927 | |
|  |  |  |  |  |  |  |  |  |  |  |  | -0.252 | 1.077 | -0.108 | 1.962 |
|  |  |  |  |  |  |  |  |  |  |  |  | 0.224 | | 0.079 | |
| ***MAPT*** | **PS** |  |  |  |  |  |  |  |  |  |  |  |  | 0.515 | |
|  |  |  |  |  |  |  |  |  |  |  |  |  |  | -0.440 | 1.469 |
|  |  |  |  |  |  |  |  |  |  |  |  |  |  | 0.291 | |
|  | **S** |  |  |  |  |  |  |  |  |  |  |  |  |  |  |
|  |  |  |  |  |  |  |  |  |  |  |  |  |  |  |  |
|  |  |  |  |  |  |  |  |  |  |  |  |  |  |  |  |

**c) Complexin-2**

|  |  | **Controls** | | ***C9orf72*** | | | | ***GRN*** | | | | ***MAPT*** | | | |
| --- | --- | --- | --- | --- | --- | --- | --- | --- | --- | --- | --- | --- | --- | --- | --- |
|  |  |  |  | **PS** | | **S** | | **PS** | | **S** | | **PS** | | **S** | |
| **controls** | |  | | 0.087 | | -0.014 | | 0.005 | | -0.086 | | 0.061 | | 0.061 | |
|  |  |  | | 0.007 | 0.166 | -0.103 | 0.074 | -0.055 | 0.064 | -0.175 | 0.002 | 0.001 | 0.121 | -0.046 | 0.169 |
|  |  |  | | **0.032** | | 0.752 | | 0.880 | | 0.056 | | **0.046** | | 0.265 | |
| **C9orf72** | **PS** |  |  |  |  | -0.101 | | -0.082 | | -0.173 | | -0.025 | | -0.025 | |
|  |  |  |  |  |  | -0.222 | 0.020 | -0.169 | 0.005 | -0.296 | -0.050 | -0.112 | 0.061 | -0.160 | 0.109 |
|  |  |  |  |  |  | 0.102 | | 0.066 | | **0.006** | | 0.564 | | 0.711 | |
|  | **S** |  |  |  |  |  |  | 0.019 | | -0.072 | | 0.075 | | 0.076 | |
|  |  |  |  |  |  |  |  | -0.079 | 0.117 | -0.180 | 0.037 | -0.027 | 0.178 | -0.045 | 0.196 |
|  |  |  |  |  |  |  |  | 0.705 | | 0.194 | | 0.149 | | 0.220 | |
| ***GRN*** | **PS** |  |  |  |  |  |  |  |  | -0.091 | | 0.057 | | 0.057 | |
|  |  |  |  |  |  |  |  |  |  | -0.189 | 0.008 | -0.018 | 0.131 | -0.059 | 0.173 |
|  |  |  |  |  |  |  |  |  |  | 0.071 | | 0.138 | | 0.339 | |
|  | **S** |  |  |  |  |  |  |  |  |  |  | 0.147 | | 0.147 | |
|  |  |  |  |  |  |  |  |  |  |  |  | 0.045 | 0.250 | 0.024 | 0.271 |
|  |  |  |  |  |  |  |  |  |  |  |  | **0.005** | | **0.019** | |
| ***MAPT*** | **PS** |  |  |  |  |  |  |  |  |  |  |  |  | 0.000 | |
|  |  |  |  |  |  |  |  |  |  |  |  |  |  | -0.121 | 0.121 |
|  |  |  |  |  |  |  |  |  |  |  |  |  |  | 0.998 | |
|  | **S** |  |  |  |  |  |  |  |  |  |  |  |  |  |  |
|  |  |  |  |  |  |  |  |  |  |  |  |  |  |  |  |
|  |  |  |  |  |  |  |  |  |  |  |  |  |  |  |  |

**d) beta-synuclein**

|  |  | **Controls** | | ***C9orf72*** | | | | ***GRN*** | | | | ***MAPT*** | | | |
| --- | --- | --- | --- | --- | --- | --- | --- | --- | --- | --- | --- | --- | --- | --- | --- |
|  |  |  |  | **PS** | | **S** | | **PS** | | **S** | | **PS** | | **S** | |
| **controls** | |  | | 0.007 | | -0.007 | | -0.001 | | 0.018 | | 0.004 | | 0.081 | |
|  |  |  | | -0.024 | 0.038 | -0.045 | 0.031 | -0.028 | 0.026 | -0.038 | 0.073 | -0.024 | 0.032 | 0.018 | 0.145 |
|  |  |  | | 0.650 | | 0.711 | | 0.967 | | 0.528 | | 0.778 | | **0.012** | |
| **C9orf72** | **PS** |  |  |  |  | -0.014 | | -0.008 | | 0.011 | | -0.003 | | 0.074 | |
|  |  |  |  |  |  | -0.057 | 0.028 | -0.040 | 0.024 | -0.048 | 0.069 | -0.035 | 0.029 | 0.009 | 0.139 |
|  |  |  |  |  |  | 0.513 | | 0.637 | | 0.720 | | 0.850 | | **0.025** | |
|  | **S** |  |  |  |  |  |  | 0.007 | | 0.025 | | 0.011 | | 0.088 | |
|  |  |  |  |  |  |  |  | -0.032 | 0.045 | -0.035 | 0.085 | -0.029 | 0.051 | 0.024 | 0.153 |
|  |  |  |  |  |  |  |  | 0.738 | | 0.417 | | 0.586 | | **0.007** | |
| ***GRN*** | **PS** |  |  |  |  |  |  |  |  | 0.018 | | 0.005 | | 0.082 | |
|  |  |  |  |  |  |  |  |  |  | -0.037 | 0.074 | -0.024 | 0.033 | 0.019 | 0.145 |
|  |  |  |  |  |  |  |  |  |  | 0.512 | | 0.753 | | **0.011** | |
|  | **S** |  |  |  |  |  |  |  |  |  |  | -0.014 | | 0.063 | |
|  |  |  |  |  |  |  |  |  |  |  |  | -0.071 | 0.044 | -0.014 | 0.141 |
|  |  |  |  |  |  |  |  |  |  |  |  | 0.639 | | 0.109 | |
| ***MAPT*** | **PS** |  |  |  |  |  |  |  |  |  |  |  |  | 0.077 | |
|  |  |  |  |  |  |  |  |  |  |  |  |  |  | 0.013 | 0.142 |
|  |  |  |  |  |  |  |  |  |  |  |  |  |  | **0.019** | |
|  | **S** |  |  |  |  |  |  |  |  |  |  |  |  |  |  |
|  |  |  |  |  |  |  |  |  |  |  |  |  |  |  |  |
|  |  |  |  |  |  |  |  |  |  |  |  |  |  |  |  |

**e) gamma-synuclein**

|  |  | **Controls** | | ***C9orf72*** | | | | ***GRN*** | | | | ***MAPT*** | | | |
| --- | --- | --- | --- | --- | --- | --- | --- | --- | --- | --- | --- | --- | --- | --- | --- |
|  |  |  |  | **PS** | | **S** | | **PS** | | **S** | | **PS** | | **S** | |
| **controls** | |  | | 0.014 | | -0.051 | | 0.022 | | -0.008 | | 0.046 | | 0.195 | |
|  |  |  | | -0.044 | 0.072 | -0.133 | 0.032 | -0.045 | 0.089 | -0.111 | 0.096 | -0.030 | 0.122 | 0.032 | 0.358 |
|  |  |  | | 0.632 | | 0.226 | | 0.524 | | 0.883 | | 0.234 | | **0.019** | |
| **C9orf72** | **PS** |  |  |  |  | -0.065 | | 0.008 | | -0.022 | | 0.032 | | 0.181 | |
|  |  |  |  |  |  | -0.158 | 0.028 | -0.069 | 0.084 | -0.133 | 0.089 | -0.051 | 0.115 | 0.016 | 0.346 |
|  |  |  |  |  |  | 0.169 | | 0.843 | | 0.699 | | 0.448 | | **0.031** | |
|  | **S** |  |  |  |  |  |  | 0.073 | | 0.043 | | 0.097 | | 0.246 | |
|  |  |  |  |  |  |  |  | -0.020 | 0.165 | -0.073 | 0.159 | -0.006 | 0.200 | 0.078 | 0.414 |
|  |  |  |  |  |  |  |  | 0.122 | | 0.466 | | 0.064 | | **0.004** | |
| ***GRN*** | **PS** |  |  |  |  |  |  |  |  | -0.030 | | 0.024 | | 0.173 | |
|  |  |  |  |  |  |  |  |  |  | -0.143 | 0.084 | -0.064 | 0.113 | 0.007 | 0.340 |
|  |  |  |  |  |  |  |  |  |  | 0.609 | | 0.591 | | **0.041** | |
|  | **S** |  |  |  |  |  |  |  |  |  |  | 0.054 | | 0.203 | |
|  |  |  |  |  |  |  |  |  |  |  |  | -0.068 | 0.176 | 0.023 | 0.383 |
|  |  |  |  |  |  |  |  |  |  |  |  | 0.385 | | **0.027** | |
| ***MAPT*** | **PS** |  |  |  |  |  |  |  |  |  |  |  |  | 0.149 | |
|  |  |  |  |  |  |  |  |  |  |  |  |  |  | -0.025 | 0.323 |
|  |  |  |  |  |  |  |  |  |  |  |  |  |  | 0.093 | |
|  | **S** |  |  |  |  |  |  |  |  |  |  |  |  |  |  |
|  |  |  |  |  |  |  |  |  |  |  |  |  |  |  |  |
|  |  |  |  |  |  |  |  |  |  |  |  |  |  |  |  |

**f) 14-3-3 eta**

|  |  | **Controls** | | ***C9orf72*** | | | | ***GRN*** | | | | ***MAPT*** | | | |
| --- | --- | --- | --- | --- | --- | --- | --- | --- | --- | --- | --- | --- | --- | --- | --- |
|  |  |  |  | **PS** | | **S** | | **PS** | | **S** | | **PS** | | **S** | |
| **controls** | |  | | 0.001 | | 0.001 | | 0.001 | | 0.002 | | 0.000 | | 0.005 | |
|  |  |  | | -0.001 | 0.002 | -0.003 | 0.005 | 0.000 | 0.003 | -0.001 | 0.005 | -0.002 | 0.002 | 0.001 | 0.009 |
|  |  |  | | 0.310 | | 0.703 | | 0.141 | | 0.262 | | 0.831 | | **0.013** | |
| **C9orf72** | **PS** |  |  |  |  | 0.000 | | 0.000 | | 0.001 | | -0.001 | | 0.004 | |
|  |  |  |  |  |  | -0.004 | 0.004 | -0.001 | 0.002 | -0.002 | 0.005 | -0.002 | 0.001 | 0.000 | 0.008 |
|  |  |  |  |  |  | 0.997 | | 0.561 | | 0.453 | | 0.524 | | **0.029** | |
|  | **S** |  |  |  |  |  |  | 0.000 | | 0.001 | | -0.001 | | 0.004 | |
|  |  |  |  |  |  |  |  | -0.003 | 0.004 | -0.003 | 0.006 | -0.005 | 0.003 | -0.001 | 0.009 |
|  |  |  |  |  |  |  |  | 0.815 | | 0.575 | | 0.778 | | 0.086 | |
| ***GRN*** | **PS** |  |  |  |  |  |  |  |  | 0.001 | | -0.001 | | 0.004 | |
|  |  |  |  |  |  |  |  |  |  | -0.003 | 0.004 | -0.003 | 0.001 | 0.000 | 0.008 |
|  |  |  |  |  |  |  |  |  |  | 0.645 | | 0.264 | | 0.051 | |
|  | **S** |  |  |  |  |  |  |  |  |  |  | -0.002 | | 0.003 | |
|  |  |  |  |  |  |  |  |  |  |  |  | -0.005 | 0.002 | -0.001 | 0.008 |
|  |  |  |  |  |  |  |  |  |  |  |  | 0.311 | | 0.186 | |
| ***MAPT*** | **PS** |  |  |  |  |  |  |  |  |  |  |  |  | 0.005 | |
|  |  |  |  |  |  |  |  |  |  |  |  |  |  | 0.001 | 0.009 |
|  |  |  |  |  |  |  |  |  |  |  |  |  |  | **0.017** | |
|  | **S** |  |  |  |  |  |  |  |  |  |  |  |  |  |  |
|  |  |  |  |  |  |  |  |  |  |  |  |  |  |  |  |
|  |  |  |  |  |  |  |  |  |  |  |  |  |  |  |  |

**g) 14-3-3 epsilon**

|  |  | **Controls** | | ***C9orf72*** | | | | ***GRN*** | | | | ***MAPT*** | | | |
| --- | --- | --- | --- | --- | --- | --- | --- | --- | --- | --- | --- | --- | --- | --- | --- |
|  |  |  |  | **PS** | | **S** | | **PS** | | **S** | | **PS** | | **S** | |
| **controls** | |  | | 0.022 | | -0.020 | | 0.031 | | 0.086 | | 0.010 | | 0.069 | |
|  |  |  | | -0.026 | 0.070 | -0.108 | 0.069 | -0.019 | 0.081 | -0.022 | 0.194 | -0.048 | 0.068 | -0.058 | 0.195 |
|  |  |  | | 0.373 | | 0.664 | | 0.220 | | 0.117 | | 0.733 | | 0.288 | |
| **C9orf72** | **PS** |  |  |  |  | -0.042 | | 0.009 | | 0.064 | | -0.012 | | 0.047 | |
|  |  |  |  |  |  | -0.116 | 0.033 | -0.029 | 0.048 | -0.031 | 0.160 | -0.063 | 0.040 | -0.071 | 0.165 |
|  |  |  |  |  |  | 0.275 | | 0.629 | | 0.187 | | 0.654 | | 0.437 | |
|  | **S** |  |  |  |  |  |  | 0.051 | | 0.106 | | 0.030 | | 0.088 | |
|  |  |  |  |  |  |  |  | -0.018 | 0.120 | 0.012 | 0.199 | -0.057 | 0.117 | -0.032 | 0.208 |
|  |  |  |  |  |  |  |  | 0.145 | | **0.026** | | 0.501 | | 0.149 | |
| ***GRN*** | **PS** |  |  |  |  |  |  |  |  | 0.055 | | -0.021 | | 0.037 | |
|  |  |  |  |  |  |  |  |  |  | -0.037 | 0.147 | -0.074 | 0.032 | -0.080 | 0.155 |
|  |  |  |  |  |  |  |  |  |  | 0.244 | | 0.431 | | 0.532 | |
|  | **S** |  |  |  |  |  |  |  |  |  |  | -0.076 | | -0.018 | |
|  |  |  |  |  |  |  |  |  |  |  |  | -0.181 | 0.029 | -0.155 | 0.120 |
|  |  |  |  |  |  |  |  |  |  |  |  | 0.156 | | 0.803 | |
| ***MAPT*** | **PS** |  |  |  |  |  |  |  |  |  |  |  |  | 0.059 | |
|  |  |  |  |  |  |  |  |  |  |  |  |  |  | -0.068 | 0.185 |
|  |  |  |  |  |  |  |  |  |  |  |  |  |  | 0.365 | |
|  | **S** |  |  |  |  |  |  |  |  |  |  |  |  |  |  |
|  |  |  |  |  |  |  |  |  |  |  |  |  |  |  |  |
|  |  |  |  |  |  |  |  |  |  |  |  |  |  |  |  |

**h) 14-3-3 zeta/delta**

|  |  | **Controls** | | ***C9orf72*** | | | | ***GRN*** | | | | ***MAPT*** | | | |
| --- | --- | --- | --- | --- | --- | --- | --- | --- | --- | --- | --- | --- | --- | --- | --- |
|  |  |  |  | **PS** | | **S** | | **PS** | | **S** | | **PS** | | **S** | |
| **controls** | |  | | -0.136 | | -0.310 | | -0.194 | | -0.163 | | -0.082 | | 0.332 | |
|  |  |  | | -0.537 | 0.264 | -1.243 | 0.622 | -0.661 | 0.273 | -1.138 | 0.813 | -0.484 | 0.321 | -0.607 | 1.272 |
|  |  |  | | 0.504 | | 0.514 | | 0.416 | | 0.744 | | 0.691 | | 0.488 | |
| **C9orf72** | **PS** |  |  |  |  | -0.174 | | -0.057 | | -0.026 | | 0.055 | | 0.469 | |
|  |  |  |  |  |  | -0.763 | 0.415 | -0.255 | 0.140 | -0.666 | 0.613 | -0.152 | 0.261 | -0.155 | 1.093 |
|  |  |  |  |  |  | 0.562 | | 0.570 | | 0.936 | | 0.603 | | 0.141 | |
|  | **S** |  |  |  |  |  |  | 0.117 | | 0.148 | | 0.229 | | 0.643 | |
|  |  |  |  |  |  |  |  | -0.401 | 0.635 | -0.168 | 0.463 | -0.389 | 0.847 | 0.264 | 1.022 |
|  |  |  |  |  |  |  |  | 0.659 | | 0.359 | | 0.468 | | **0.001** | |
| ***GRN*** | **PS** |  |  |  |  |  |  |  |  | 0.031 | | 0.112 | | 0.526 | |
|  |  |  |  |  |  |  |  |  |  | -0.544 | 0.606 | -0.126 | 0.351 | -0.038 | 1.091 |
|  |  |  |  |  |  |  |  |  |  | 0.916 | | 0.356 | | 0.068 | |
|  | **S** |  |  |  |  |  |  |  |  |  |  | 0.081 | | 0.495 | |
|  |  |  |  |  |  |  |  |  |  |  |  | -0.583 | 0.746 | 0.063 | 0.928 |
|  |  |  |  |  |  |  |  |  |  |  |  | 0.811 | | **0.025** | |
| ***MAPT*** | **PS** |  |  |  |  |  |  |  |  |  |  |  |  | 0.414 | |
|  |  |  |  |  |  |  |  |  |  |  |  |  |  | -0.239 | 1.067 |
|  |  |  |  |  |  |  |  |  |  |  |  |  |  | 0.214 | |
|  | **S** |  |  |  |  |  |  |  |  |  |  |  |  |  |  |
|  |  |  |  |  |  |  |  |  |  |  |  |  |  |  |  |
|  |  |  |  |  |  |  |  |  |  |  |  |  |  |  |  |

**i) neurogranin**

|  |  | **Controls** | | ***C9orf72*** | | | | ***GRN*** | | | | ***MAPT*** | | | |
| --- | --- | --- | --- | --- | --- | --- | --- | --- | --- | --- | --- | --- | --- | --- | --- |
|  |  |  |  | **PS** | | **S** | | **PS** | | **S** | | **PS** | | **S** | |
| **controls** | |  | | 0.002 | | -0.002 | | 0.001 | | -0.001 | | 0.003 | | 0.026 | |
|  |  |  | | -0.004 | 0.008 | -0.011 | 0.007 | -0.006 | 0.007 | -0.011 | 0.008 | -0.003 | 0.009 | 0.008 | 0.043 |
|  |  |  | | 0.570 | | 0.703 | | 0.818 | | 0.776 | | 0.346 | | **0.004** | |
| **C9orf72** | **PS** |  |  |  |  | -0.004 | | -0.001 | | -0.003 | | 0.001 | | 0.024 | |
|  |  |  |  |  |  | -0.013 | 0.006 | -0.008 | 0.006 | -0.014 | 0.007 | -0.006 | 0.008 | 0.007 | 0.041 |
|  |  |  |  |  |  | 0.482 | | 0.784 | | 0.552 | | 0.733 | | **0.007** | |
|  | **S** |  |  |  |  |  |  | 0.003 | | 0.000 | | 0.005 | | 0.027 | |
|  |  |  |  |  |  |  |  | -0.007 | 0.012 | -0.011 | 0.012 | -0.005 | 0.015 | 0.009 | 0.045 |
|  |  |  |  |  |  |  |  | 0.613 | | 0.953 | | 0.353 | | **0.003** | |
| ***GRN*** | **PS** |  |  |  |  |  |  |  |  | -0.002 | | 0.002 | | 0.025 | |
|  |  |  |  |  |  |  |  |  |  | -0.013 | 0.008 | -0.005 | 0.010 | 0.007 | 0.042 |
|  |  |  |  |  |  |  |  |  |  | 0.686 | | 0.554 | | **0.005** | |
|  | **S** |  |  |  |  |  |  |  |  |  |  | 0.004 | | 0.027 | |
|  |  |  |  |  |  |  |  |  |  |  |  | -0.006 | 0.015 | 0.008 | 0.045 |
|  |  |  |  |  |  |  |  |  |  |  |  | 0.423 | | **0.004** | |
| ***MAPT*** | **PS** |  |  |  |  |  |  |  |  |  |  |  |  | 0.023 | |
|  |  |  |  |  |  |  |  |  |  |  |  |  |  | 0.005 | 0.040 |
|  |  |  |  |  |  |  |  |  |  |  |  |  |  | **0.013** | |
|  | **S** |  |  |  |  |  |  |  |  |  |  |  |  |  |  |
|  |  |  |  |  |  |  |  |  |  |  |  |  |  |  |  |
|  |  |  |  |  |  |  |  |  |  |  |  |  |  |  |  |

**j) Rab GDI alpha**

|  |  | **Controls** | | ***C9orf72*** | | | | ***GRN*** | | | | ***MAPT*** | | | |
| --- | --- | --- | --- | --- | --- | --- | --- | --- | --- | --- | --- | --- | --- | --- | --- |
|  |  |  |  | **PS** | | **S** | | **PS** | | **S** | | **PS** | | **S** | |
| **controls** | |  | | 0.011 | | 0.016 | | 0.002 | | 0.003 | | 0.016 | | 0.071 | |
|  |  |  | | -0.007 | 0.030 | -0.013 | 0.044 | -0.017 | 0.022 | -0.030 | 0.037 | -0.002 | 0.034 | 0.027 | 0.115 |
|  |  |  | | 0.228 | | 0.272 | | 0.809 | | 0.840 | | 0.081 | | **0.002** | |
| **C9orf72** | **PS** |  |  |  |  | 0.005 | | -0.009 | | -0.008 | | 0.004 | | 0.060 | |
|  |  |  |  |  |  | -0.027 | 0.036 | -0.032 | 0.013 | -0.045 | 0.029 | -0.016 | 0.025 | 0.014 | 0.105 |
|  |  |  |  |  |  | 0.780 | | 0.429 | | 0.672 | | 0.676 | | **0.011** | |
|  | **S** |  |  |  |  |  |  | -0.014 | | -0.012 | | 0.000 | | 0.055 | |
|  |  |  |  |  |  |  |  | -0.043 | 0.016 | -0.052 | 0.027 | -0.031 | 0.031 | 0.008 | 0.103 |
|  |  |  |  |  |  |  |  | 0.372 | | 0.539 | | 0.994 | | **0.023** | |
| ***GRN*** | **PS** |  |  |  |  |  |  |  |  | 0.001 | | 0.013 | | 0.069 | |
|  |  |  |  |  |  |  |  |  |  | -0.034 | 0.036 | -0.008 | 0.035 | 0.024 | 0.113 |
|  |  |  |  |  |  |  |  |  |  | 0.950 | | 0.214 | | **0.003** | |
|  | **S** |  |  |  |  |  |  |  |  |  |  | 0.012 | | 0.068 | |
|  |  |  |  |  |  |  |  |  |  |  |  | -0.024 | 0.049 | 0.016 | 0.119 |
|  |  |  |  |  |  |  |  |  |  |  |  | 0.506 | | **0.010** | |
| ***MAPT*** | **PS** |  |  |  |  |  |  |  |  |  |  |  |  | 0.055 | |
|  |  |  |  |  |  |  |  |  |  |  |  |  |  | 0.009 | 0.102 |
|  |  |  |  |  |  |  |  |  |  |  |  |  |  | **0.020** | |
|  | **S** |  |  |  |  |  |  |  |  |  |  |  |  |  |  |
|  |  |  |  |  |  |  |  |  |  |  |  |  |  |  |  |
|  |  |  |  |  |  |  |  |  |  |  |  |  |  |  |  |

**k) Syntaxin-1B**

|  |  | **Controls** | | ***C9orf72*** | | | | ***GRN*** | | | | ***MAPT*** | | | |
| --- | --- | --- | --- | --- | --- | --- | --- | --- | --- | --- | --- | --- | --- | --- | --- |
|  |  |  |  | **PS** | | **S** | | **PS** | | **S** | | **PS** | | **S** | |
| **controls** | |  | | 0.000 | | -0.001 | | 0.001 | | -0.003 | | 0.001 | | 0.007 | |
|  |  |  | | -0.002 | 0.003 | -0.004 | 0.003 | -0.002 | 0.003 | -0.007 | 0.002 | -0.002 | 0.004 | 0.001 | 0.013 |
|  |  |  | | 0.826 | | 0.778 | | 0.598 | | 0.213 | | 0.516 | | **0.016** | |
| **C9orf72** | **PS** |  |  |  |  | -0.001 | | 0.000 | | -0.003 | | 0.001 | | 0.007 | |
|  |  |  |  |  |  | -0.005 | 0.003 | -0.003 | 0.004 | -0.008 | 0.002 | -0.002 | 0.004 | 0.001 | 0.013 |
|  |  |  |  |  |  | 0.698 | | 0.786 | | 0.207 | | 0.705 | | **0.024** | |
|  | **S** |  |  |  |  |  |  | 0.001 | | -0.002 | | 0.001 | | 0.008 | |
|  |  |  |  |  |  |  |  | -0.003 | 0.005 | -0.007 | 0.003 | -0.003 | 0.006 | 0.002 | 0.014 |
|  |  |  |  |  |  |  |  | 0.527 | | 0.409 | | 0.505 | | **0.013** | |
| ***GRN*** | **PS** |  |  |  |  |  |  |  |  | -0.003 | | 0.000 | | 0.007 | |
|  |  |  |  |  |  |  |  |  |  | -0.008 | 0.001 | -0.003 | 0.003 | 0.001 | 0.013 |
|  |  |  |  |  |  |  |  |  |  | 0.133 | | 0.928 | | **0.031** | |
|  | **S** |  |  |  |  |  |  |  |  |  |  | 0.004 | | 0.010 | |
|  |  |  |  |  |  |  |  |  |  |  |  | -0.001 | 0.008 | 0.003 | 0.017 |
|  |  |  |  |  |  |  |  |  |  |  |  | 0.134 | | **0.003** | |
| ***MAPT*** | **PS** |  |  |  |  |  |  |  |  |  |  |  |  | 0.006 | |
|  |  |  |  |  |  |  |  |  |  |  |  |  |  | 0.000 | 0.013 |
|  |  |  |  |  |  |  |  |  |  |  |  |  |  | **0.045** | |
|  | **S** |  |  |  |  |  |  |  |  |  |  |  |  |  |  |
|  |  |  |  |  |  |  |  |  |  |  |  |  |  |  |  |
|  |  |  |  |  |  |  |  |  |  |  |  |  |  |  |  |

**l) Syntaxin-7**

|  |  | **Controls** | | ***C9orf72*** | | | | ***GRN*** | | | | ***MAPT*** | | | |
| --- | --- | --- | --- | --- | --- | --- | --- | --- | --- | --- | --- | --- | --- | --- | --- |
|  |  |  |  | **PS** | | **S** | | **PS** | | **S** | | **PS** | | **S** | |
| **controls** | |  | | -0.003 | | -0.024 | | 0.015 | | -0.005 | | 0.003 | | 0.056 | |
|  |  |  | | -0.025 | 0.019 | -0.053 | 0.005 | -0.009 | 0.039 | -0.041 | 0.031 | -0.020 | 0.027 | 0.000 | 0.113 |
|  |  |  | | 0.815 | | 0.102 | | 0.228 | | 0.799 | | 0.784 | | **0.049** | |
| **C9orf72** | **PS** |  |  |  |  | -0.021 | | 0.018 | | -0.002 | | 0.006 | | 0.059 | |
|  |  |  |  |  |  | -0.054 | 0.011 | -0.009 | 0.045 | -0.041 | 0.036 | -0.019 | 0.031 | 0.001 | 0.117 |
|  |  |  |  |  |  | 0.193 | | 0.201 | | 0.916 | | 0.649 | | **0.045** | |
|  | **S** |  |  |  |  |  |  | 0.039 | | 0.019 | | 0.027 | | 0.080 | |
|  |  |  |  |  |  |  |  | 0.007 | 0.071 | -0.021 | 0.060 | -0.006 | 0.061 | 0.024 | 0.136 |
|  |  |  |  |  |  |  |  | **0.016** | | 0.348 | | 0.110 | | **0.005** | |
| ***GRN*** | **PS** |  |  |  |  |  |  |  |  | -0.020 | | -0.012 | | 0.041 | |
|  |  |  |  |  |  |  |  |  |  | -0.059 | 0.019 | -0.039 | 0.016 | -0.016 | 0.099 |
|  |  |  |  |  |  |  |  |  |  | 0.322 | | 0.404 | | 0.158 | |
|  | **S** |  |  |  |  |  |  |  |  |  |  | 0.008 | | 0.061 | |
|  |  |  |  |  |  |  |  |  |  |  |  | -0.031 | 0.047 | 0.000 | 0.122 |
|  |  |  |  |  |  |  |  |  |  |  |  | 0.691 | | **0.049** | |
| ***MAPT*** | **PS** |  |  |  |  |  |  |  |  |  |  |  |  | 0.053 | |
|  |  |  |  |  |  |  |  |  |  |  |  |  |  | -0.006 | 0.112 |
|  |  |  |  |  |  |  |  |  |  |  |  |  |  | 0.076 | |
|  | **S** |  |  |  |  |  |  |  |  |  |  |  |  |  |  |
|  |  |  |  |  |  |  |  |  |  |  |  |  |  |  |  |
|  |  |  |  |  |  |  |  |  |  |  |  |  |  |  |  |

**m) PEBP-1(NRPTSISWDGLDSGK)**

|  |  | **Controls** | | ***C9orf72*** | | | | ***GRN*** | | | | ***MAPT*** | | | | |
| --- | --- | --- | --- | --- | --- | --- | --- | --- | --- | --- | --- | --- | --- | --- | --- | --- |
|  |  |  |  | **PS** | | **S** | | **PS** | | **S** | | **PS** | | **S** | | |
| **controls** | |  | | 0.567 | | 0.337 | | 0.323 | | -0.520 | | 0.947 | | 4.612 | | |
|  |  |  | | -0.959 | 2.094 | -2.317 | 2.992 | -1.299 | 1.945 | -3.444 | 2.404 | -0.598 | 2.491 | 1.556 | 7.667 |  |
|  |  |  | | 0.466 | | 0.803 | | 0.696 | | 0.727 | | 0.229 | | **0.003** | | |
| **C9orf72** | **PS** |  |  |  |  | -0.230 | | -0.244 | | -1.087 | | 0.380 | | 4.044 | |  |
|  |  |  |  |  |  | -3.054 | 2.594 | -2.087 | 1.598 | -4.169 | 1.994 | -1.363 | 2.123 | 0.887 | 7.201 |  |
|  |  |  |  |  |  | 0.873 | | 0.795 | | 0.489 | | 0.670 | | **0.012** | |  |
|  | **S** |  |  |  |  |  |  | -0.014 | | -0.857 | | 0.609 | | 4.274 | |  |
|  |  |  |  |  |  |  |  | -2.736 | 2.707 | -4.520 | 2.805 | -2.248 | 3.467 | 0.691 | 7.857 |  |
|  |  |  |  |  |  |  |  | 0.992 | | 0.646 | | 0.676 | | **0.019** | |  |
| ***GRN*** | **PS** |  |  |  |  |  |  |  |  | -0.843 | | 0.624 | | 4.289 | |  |
|  |  |  |  |  |  |  |  |  |  | -3.899 | 2.213 | -1.201 | 2.449 | 1.182 | 7.396 |  |
|  |  |  |  |  |  |  |  |  |  | 0.589 | | 0.503 | | **0.007** | |  |
|  | **S** |  |  |  |  |  |  |  |  |  |  | 1.467 | | 5.132 | |  |
|  |  |  |  |  |  |  |  |  |  |  |  | -1.681 | 4.615 | 1.196 | 9.067 |  |
|  |  |  |  |  |  |  |  |  |  |  |  | 0.361 | | **0.011** | |  |
| ***MAPT*** | **PS** |  |  |  |  |  |  |  |  |  |  |  |  | 3.665 | |  |
|  |  |  |  |  |  |  |  |  |  |  |  |  |  | 0.382 | 6.948 |  |
|  |  |  |  |  |  |  |  |  |  |  |  |  |  | **0.029** | |  |
|  | **S** |  |  |  |  |  |  |  |  |  |  |  |  |  |  |  |
|  |  |  |  |  |  |  |  |  |  |  |  |  |  |  |  |  |
|  |  |  |  |  |  |  |  |  |  |  |  |  |  |  |  |  |

**n) PEBP-1 (LYEQLSGK)**

|  |  | **Controls** | | ***C9orf72*** | | | | ***GRN*** | | | | ***MAPT*** | | | |
| --- | --- | --- | --- | --- | --- | --- | --- | --- | --- | --- | --- | --- | --- | --- | --- |
|  |  |  |  | **PS** | | **S** | | **PS** | | **S** | | **PS** | | **S** | |
| **controls** | |  | | 0.055 | | -0.041 | | -0.007 | | 0.035 | | 0.080 | | 0.432 | |
|  |  |  | | -0.080 | 0.191 | -0.333 | 0.252 | -0.186 | 0.172 | -0.275 | 0.345 | -0.092 | 0.252 | 0.140 | 0.723 |
|  |  |  | | 0.424 | | 0.784 | | 0.940 | | 0.823 | | 0.363 | | **0.004** | |
| **C9orf72** | **PS** |  |  |  |  | -0.096 | | -0.062 | | -0.020 | | 0.024 | | 0.376 | |
|  |  |  |  |  |  | -0.381 | 0.188 | -0.243 | 0.118 | -0.327 | 0.287 | -0.151 | 0.200 | 0.089 | 0.664 |
|  |  |  |  |  |  | 0.506 | | 0.499 | | 0.898 | | 0.785 | | **0.010** | |
|  | **S** |  |  |  |  |  |  | 0.034 | | 0.076 | | 0.121 | | 0.473 | |
|  |  |  |  |  |  |  |  | -0.263 | 0.331 | -0.301 | 0.453 | -0.184 | 0.426 | 0.123 | 0.822 |
|  |  |  |  |  |  |  |  | 0.822 | | 0.691 | | 0.437 | | **0.008** | |
| ***GRN*** | **PS** |  |  |  |  |  |  |  |  | 0.042 | | 0.087 | | 0.439 | |
|  |  |  |  |  |  |  |  |  |  | -0.276 | 0.361 | -0.121 | 0.295 | 0.144 | 0.734 |
|  |  |  |  |  |  |  |  |  |  | 0.795 | | 0.414 | | **0.004** | |
|  | **S** |  |  |  |  |  |  |  |  |  |  | 0.045 | | 0.396 | |
|  |  |  |  |  |  |  |  |  |  |  |  | -0.281 | 0.370 | 0.025 | 0.768 |
|  |  |  |  |  |  |  |  |  |  |  |  | 0.788 | | **0.036** | |
| ***MAPT*** | **PS** |  |  |  |  |  |  |  |  |  |  |  |  | 0.352 | |
|  |  |  |  |  |  |  |  |  |  |  |  |  |  | 0.038 | 0.666 |
|  |  |  |  |  |  |  |  |  |  |  |  |  |  | **0.028** | |
|  | **S** |  |  |  |  |  |  |  |  |  |  |  |  |  |  |
|  |  |  |  |  |  |  |  |  |  |  |  |  |  |  |  |
|  |  |  |  |  |  |  |  |  |  |  |  |  |  |  |  |

**o) NPTXR (NNYMYAR)**

|  |  | **Controls** | | ***C9orf72*** | | | | ***GRN*** | | | | ***MAPT*** | | | |
| --- | --- | --- | --- | --- | --- | --- | --- | --- | --- | --- | --- | --- | --- | --- | --- |
|  |  |  |  | **PS** | | **S** | | **PS** | | **S** | | **PS** | | **S** | |
| **controls** | |  | | 0.022 | | -1.020 | | 0.173 | | -0.856 | | -0.057 | | -0.482 | |
|  |  |  | | -0.458 | 0.502 | -1.526 | -0.514 | -0.337 | 0.683 | -1.538 | -0.174 | -0.587 | 0.474 | -1.183 | 0.219 |
|  |  |  | | 0.929 | | **<0.001** | | 0.505 | | **0.014** | | 0.835 | | 0.178 | |
| **C9orf72** | **PS** |  |  |  |  | -1.042 | | 0.152 | | -0.878 | | -0.078 | | -0.504 | |
|  |  |  |  |  |  | -1.636 | -0.448 | -0.435 | 0.739 | -1.634 | -0.121 | -0.668 | 0.511 | -1.264 | 0.257 |
|  |  |  |  |  |  | **0.001** | | 0.613 | | **0.023** | | 0.795 | | 0.194 | |
|  | **S** |  |  |  |  |  |  | 1.194 | | 0.164 | | 0.964 | | 0.538 | |
|  |  |  |  |  |  |  |  | 0.571 | 1.816 | -0.543 | 0.872 | 0.309 | 1.618 | -0.192 | 1.269 |
|  |  |  |  |  |  |  |  | **<0.001** | | 0.649 | | **0.004** | | 0.149 | |
| ***GRN*** | **PS** |  |  |  |  |  |  |  |  | -1.029 | | -0.230 | | -0.655 | |
|  |  |  |  |  |  |  |  |  |  | -1.813 | -0.245 | -0.837 | 0.377 | -1.446 | 0.135 |
|  |  |  |  |  |  |  |  |  |  | **0.010** | | 0.458 | | 0.104 | |
|  | **S** |  |  |  |  |  |  |  |  |  |  | 0.799 | | 0.374 | |
|  |  |  |  |  |  |  |  |  |  |  |  | -0.009 | 1.608 | -0.520 | 1.268 |
|  |  |  |  |  |  |  |  |  |  |  |  | 0.053 | | 0.412 | |
| ***MAPT*** | **PS** |  |  |  |  |  |  |  |  |  |  |  |  | -0.425 | |
|  |  |  |  |  |  |  |  |  |  |  |  |  |  | -1.248 | 0.397 |
|  |  |  |  |  |  |  |  |  |  |  |  |  |  | 0.311 | |
|  | **S** |  |  |  |  |  |  |  |  |  |  |  |  |  |  |
|  |  |  |  |  |  |  |  |  |  |  |  |  |  |  |  |
|  |  |  |  |  |  |  |  |  |  |  |  |  |  |  |  |

**p) NPTXR (LVEAFGGATK)**

|  |  | **Controls** | | ***C9orf72*** | | | | ***GRN*** | | | | ***MAPT*** | | | |
| --- | --- | --- | --- | --- | --- | --- | --- | --- | --- | --- | --- | --- | --- | --- | --- |
|  |  |  |  | **PS** | | **S** | | **PS** | | **S** | | **PS** | | **S** | |
| **controls** | |  | | 0.355 | | -2.524 | | 0.849 | | -1.400 | | 0.151 | | -1.487 | |
|  |  |  | | -1.110 | 1.821 | -4.098 | -0.950 | -0.590 | 2.287 | -3.312 | 0.511 | -1.366 | 1.668 | -3.474 | 0.500 |
|  |  |  | | 0.634 | | **0.002** | | 0.248 | | 0.151 | | 0.845 | | 0.142 | |
| **C9orf72** | **PS** |  |  |  |  | -2.880 | | 0.493 | | -1.756 | | -0.204 | | -1.843 | |
|  |  |  |  |  |  | -4.600 | -1.160 | -1.052 | 2.038 | -3.787 | 0.276 | -1.818 | 1.410 | -3.934 | 0.249 |
|  |  |  |  |  |  | **0.001** | | 0.532 | | **0.090** | | 0.804 | | 0.084 | |
|  | **S** |  |  |  |  |  |  | 3.373 | | 1.124 | | 2.675 | | 1.037 | |
|  |  |  |  |  |  |  |  | 1.656 | 5.090 | -0.744 | 2.992 | 0.813 | 4.538 | -0.989 | 3.063 |
|  |  |  |  |  |  |  |  | **<0.001** | | 0.238 | | **0.005** | | 0.316 | |
| ***GRN*** | **PS** |  |  |  |  |  |  |  |  | -2.249 | | -0.697 | | -2.336 | |
|  |  |  |  |  |  |  |  |  |  | -4.287 | -0.210 | -2.282 | 0.887 | -4.467 | -0.204 |
|  |  |  |  |  |  |  |  |  |  | **0.031** | | 0.388 | | **0.032** | |
|  | **S** |  |  |  |  |  |  |  |  |  |  | 1.551 | | -0.087 | |
|  |  |  |  |  |  |  |  |  |  |  |  | -0.617 | 3.720 | -2.429 | 2.255 |
|  |  |  |  |  |  |  |  |  |  |  |  | 0.161 | | 0.942 | |
| ***MAPT*** | **PS** |  |  |  |  |  |  |  |  |  |  |  |  | -1.638 | |
|  |  |  |  |  |  |  |  |  |  |  |  |  |  | -3.867 | 0.590 |
|  |  |  |  |  |  |  |  |  |  |  |  |  |  | 0.150 | |
|  | **S** |  |  |  |  |  |  |  |  |  |  |  |  |  |  |
|  |  |  |  |  |  |  |  |  |  |  |  |  |  |  |  |
|  |  |  |  |  |  |  |  |  |  |  |  |  |  |  |  |

**q) NPTX1 (CESQSTLDPGAGEAR)**

|  |  | **Controls** | | ***C9orf72*** | | | | ***GRN*** | | | | ***MAPT*** | | | |
| --- | --- | --- | --- | --- | --- | --- | --- | --- | --- | --- | --- | --- | --- | --- | --- |
|  |  |  |  | **PS** | | **S** | | **PS** | | **S** | | **PS** | | **S** | |
| **controls** | |  | | -0.049 | | -0.735 | | 0.109 | | -0.654 | | -0.106 | | -0.747 | |
|  |  |  | | -0.393 | 0.295 | -1.112 | -0.358 | -0.386 | 0.604 | -1.147 | -0.160 | -0.531 | 0.319 | -1.221 | -0.272 |
|  |  |  | | 0.779 | | **<0.001** | | 0.665 | | **0.009** | | 0.625 | | **0.002** | |
| **C9orf72** | **PS** |  |  |  |  | -0.686 | | 0.158 | | -0.605 | | -0.057 | | -0.698 | |
|  |  |  |  |  |  | -1.136 | -0.235 | -0.383 | 0.700 | -1.153 | -0.056 | -0.516 | 0.402 | -1.224 | -0.171 |
|  |  |  |  |  |  | **0.003** | | 0.566 | | **0.031** | | 0.808 | | **0.009** | |
|  | **S** |  |  |  |  |  |  | 0.844 | | 0.081 | | 0.629 | | -0.012 | |
|  |  |  |  |  |  |  |  | 0.317 | 1.371 | -0.416 | 0.578 | 0.121 | 1.137 | -0.472 | 0.448 |
|  |  |  |  |  |  |  |  | **0.002** | | 0.750 | | **0.015** | | 0.959 | |
| ***GRN*** | **PS** |  |  |  |  |  |  |  |  | -0.763 | | -0.216 | | -0.856 | |
|  |  |  |  |  |  |  |  |  |  | -1.398 | -0.129 | -0.794 | 0.363 | -1.470 | -0.242 |
|  |  |  |  |  |  |  |  |  |  | **0.018** | | 0.465 | | **0.006** | |
|  | **S** |  |  |  |  |  |  |  |  |  |  | 0.548 | | -0.093 | |
|  |  |  |  |  |  |  |  |  |  |  |  | -0.063 | 1.158 | -0.660 | 0.475 |
|  |  |  |  |  |  |  |  |  |  |  |  | 0.079 | | 0.749 | |
| ***MAPT*** | **PS** |  |  |  |  |  |  |  |  |  |  |  |  | -0.640 | |
|  |  |  |  |  |  |  |  |  |  |  |  |  |  | -1.232 | -0.049 |
|  |  |  |  |  |  |  |  |  |  |  |  |  |  | **0.034** | |
|  | **S** |  |  |  |  |  |  |  |  |  |  |  |  |  |  |
|  |  |  |  |  |  |  |  |  |  |  |  |  |  |  |  |
|  |  |  |  |  |  |  |  |  |  |  |  |  |  |  |  |

**r) NPTX1 (LENLEQYSR)**

|  |  | **Controls** | | ***C9orf72*** | | | | ***GRN*** | | | | ***MAPT*** | | | |
| --- | --- | --- | --- | --- | --- | --- | --- | --- | --- | --- | --- | --- | --- | --- | --- |
|  |  |  |  | **PS** | | **S** | | **PS** | | **S** | | **PS** | | **S** | |
| **controls** | |  | | 0.000 | | -0.064 | | 0.019 | | -0.016 | | -0.006 | | -0.023 | |
|  |  |  | | -0.035 | 0.036 | -0.107 | -0.020 | -0.018 | 0.056 | -0.076 | 0.044 | -0.045 | 0.033 | -0.080 | 0.035 |
|  |  |  | | 0.978 | | **0.004** | | 0.314 | | 0.603 | | 0.760 | | 0.440 | |
| **C9orf72** | **PS** |  |  |  |  | -0.064 | | 0.019 | | -0.017 | | -0.007 | | -0.023 | |
|  |  |  |  |  |  | -0.110 | -0.018 | -0.020 | 0.057 | -0.078 | 0.045 | -0.046 | 0.033 | -0.082 | 0.036 |
|  |  |  |  |  |  | **0.006** | | 0.342 | | 0.599 | | 0.746 | | 0.444 | |
|  | **S** |  |  |  |  |  |  | 0.083 | | 0.048 | | 0.057 | | 0.041 | |
|  |  |  |  |  |  |  |  | 0.036 | 0.129 | -0.016 | 0.111 | 0.006 | 0.109 | -0.021 | 0.103 |
|  |  |  |  |  |  |  |  | **<0.001** | | 0.144 | | **0.028** | | 0.197 | |
| ***GRN*** | **PS** |  |  |  |  |  |  |  |  | -0.035 | | -0.025 | | -0.042 | |
|  |  |  |  |  |  |  |  |  |  | -0.098 | 0.028 | -0.068 | 0.017 | -0.102 | 0.018 |
|  |  |  |  |  |  |  |  |  |  | 0.272 | | 0.244 | | 0.172 | |
|  | **S** |  |  |  |  |  |  |  |  |  |  | 0.010 | | -0.007 | |
|  |  |  |  |  |  |  |  |  |  |  |  | -0.056 | 0.076 | -0.084 | 0.071 |
|  |  |  |  |  |  |  |  |  |  |  |  | 0.768 | | 0.867 | |
| ***MAPT*** | **PS** |  |  |  |  |  |  |  |  |  |  |  |  | -0.017 | |
|  |  |  |  |  |  |  |  |  |  |  |  |  |  | -0.080 | 0.047 |
|  |  |  |  |  |  |  |  |  |  |  |  |  |  | 0.610 | |
|  | **S** |  |  |  |  |  |  |  |  |  |  |  |  |  |  |
|  |  |  |  |  |  |  |  |  |  |  |  |  |  |  |  |
|  |  |  |  |  |  |  |  |  |  |  |  |  |  |  |  |

**s) NPTX2 (WPVETCEER)**

|  |  | **Controls** | | ***C9orf72*** | | | | ***GRN*** | | | | ***MAPT*** | | | |
| --- | --- | --- | --- | --- | --- | --- | --- | --- | --- | --- | --- | --- | --- | --- | --- |
|  |  |  |  | **PS** | | **S** | | **PS** | | **S** | | **PS** | | **S** | |
| **controls** | |  | | 0.035 | | -0.142 | | 0.042 | | -0.117 | | -0.011 | | -0.084 | |
|  |  |  | | -0.033 | 0.103 | -0.211 | -0.073 | -0.028 | 0.112 | -0.198 | -0.036 | -0.084 | 0.063 | -0.175 | 0.008 |
|  |  |  | | 0.312 | | **<0.001** | | 0.244 | | **0.005** | | 0.778 | | 0.074 | |
| **C9orf72** | **PS** |  |  |  |  | -0.177 | | 0.006 | | -0.152 | | -0.046 | | -0.119 | |
|  |  |  |  |  |  | -0.259 | -0.096 | -0.075 | 0.088 | -0.244 | -0.060 | -0.129 | 0.037 | -0.221 | -0.017 |
|  |  |  |  |  |  | **<0.001** | | 0.876 | | **0.001** | | 0.278 | | **0.022** | |
|  | **S** |  |  |  |  |  |  | 0.184 | | 0.026 | | 0.132 | | 0.059 | |
|  |  |  |  |  |  |  |  | 0.098 | 0.270 | -0.054 | 0.105 | 0.042 | 0.221 | -0.036 | 0.153 |
|  |  |  |  |  |  |  |  | **<0.001** | | 0.529 | | **0.004** | | 0.224 | |
| ***GRN*** | **PS** |  |  |  |  |  |  |  |  | -0.158 | | -0.052 | | -0.125 | |
|  |  |  |  |  |  |  |  |  |  | -0.256 | -0.061 | -0.136 | 0.031 | -0.231 | -0.019 |
|  |  |  |  |  |  |  |  |  |  | **0.001** | | 0.220 | | **0.020** | |
|  | **S** |  |  |  |  |  |  |  |  |  |  | 0.106 | | 0.033 | |
|  |  |  |  |  |  |  |  |  |  |  |  | 0.006 | 0.206 | -0.074 | 0.140 |
|  |  |  |  |  |  |  |  |  |  |  |  | **0.038** | | 0.545 | |
| ***MAPT*** | **PS** |  |  |  |  |  |  |  |  |  |  |  |  | -0.073 | |
|  |  |  |  |  |  |  |  |  |  |  |  |  |  | -0.182 | 0.036 |
|  |  |  |  |  |  |  |  |  |  |  |  |  |  | 0.189 | |
|  | **S** |  |  |  |  |  |  |  |  |  |  |  |  |  |  |
|  |  |  |  |  |  |  |  |  |  |  |  |  |  |  |  |
|  |  |  |  |  |  |  |  |  |  |  |  |  |  |  |  |

**t) NPTX2 (VAELEDEK)**

|  |  | **Controls** | | ***C9orf72*** | | | | ***GRN*** | | | | ***MAPT*** | | | |
| --- | --- | --- | --- | --- | --- | --- | --- | --- | --- | --- | --- | --- | --- | --- | --- |
|  |  |  |  | **PS** | | **S** | | **PS** | | **S** | | **PS** | | **S** | |
| **controls** | |  | | 0.040 | | -0.453 | | 0.085 | | -0.426 | | -0.078 | | -0.363 | |
|  |  |  | | -0.155 | 0.235 | -0.654 | -0.253 | -0.128 | 0.298 | -0.667 | -0.185 | -0.287 | 0.132 | -0.592 | -0.135 |
|  |  |  | | 0.688 | | **<0.001** | | 0.434 | | **0.001** | | 0.467 | | **0.002** | |
| **C9orf72** | **PS** |  |  |  |  | -0.493 | | 0.045 | | -0.466 | | -0.118 | | -0.403 | |
|  |  |  |  |  |  | -0.726 | -0.260 | -0.197 | 0.287 | -0.737 | -0.196 | -0.351 | 0.116 | -0.660 | -0.147 |
|  |  |  |  |  |  | **<0.001** | | 0.715 | | **0.001** | | 0.324 | | **0.002** | |
|  | **S** |  |  |  |  |  |  | 0.538 | | 0.027 | | 0.376 | | 0.090 | |
|  |  |  |  |  |  |  |  | 0.271 | 0.805 | -0.211 | 0.265 | 0.120 | 0.631 | -0.143 | 0.323 |
|  |  |  |  |  |  |  |  | **<0.001** | | 0.826 | | **0.004** | | 0.449 | |
| ***GRN*** | **PS** |  |  |  |  |  |  |  |  | -0.511 | | -0.163 | | -0.448 | |
|  |  |  |  |  |  |  |  |  |  | -0.814 | -0.209 | -0.404 | 0.079 | -0.734 | -0.163 |
|  |  |  |  |  |  |  |  |  |  | **0.001** | | 0.187 | | **0.002** | |
|  | **S** |  |  |  |  |  |  |  |  |  |  | 0.349 | | 0.063 | |
|  |  |  |  |  |  |  |  |  |  |  |  | 0.058 | 0.640 | -0.217 | 0.343 |
|  |  |  |  |  |  |  |  |  |  |  |  | **0.019** | | 0.658 | |
| ***MAPT*** | **PS** |  |  |  |  |  |  |  |  |  |  |  |  | -0.286 | |
|  |  |  |  |  |  |  |  |  |  |  |  |  |  | -0.564 | -0.007 |
|  |  |  |  |  |  |  |  |  |  |  |  |  |  | **0.044** | |
|  | **S** |  |  |  |  |  |  |  |  |  |  |  |  |  |  |
|  |  |  |  |  |  |  |  |  |  |  |  |  |  |  |  |
|  |  |  |  |  |  |  |  |  |  |  |  |  |  |  |  |
