# Supplementary material for: Differential impairment of cerebrospinal fluid synaptic biomarkers in the genetic forms of frontotemporal dementia
Source: Alzheimers Res Ther. 2022 Aug 31;14:118. doi: 10.1186/s13195-022-01042-3 (PMC9429339; doi:10.1186/s13195-022-01042-3)
Supplement: Supplementary file 2 — Additional file 2: Appendix 1. List of GENFI consortium authors. [file 13195_2022_1042_MOESM2_ESM.docx]

**Appendix 1**

***List of GENFI consortium authors***

| **Author** | **Affiliation** |
| --- | --- |
| Annabel Nelson | Department of Neurodegenerative Disease, Dementia Research Centre, UCL Queen Square Institute of Neurology, London, UK |
| Arabella Bouzigues | Department of Neurodegenerative Disease, Dementia Research Centre, UCL Queen Square Institute of Neurology, London, UK |
| Caroline V Greaves | Department of Neurodegenerative Disease, Dementia Research Centre, UCL Queen Square Institute of Neurology, London, UK |
| David Cash | Department of Neurodegenerative Disease, Dementia Research Centre, UCL Queen Square Institute of Neurology, London, UK |
| David L Thomas | Neuroimaging Analysis Centre, Department of Brain Repair and Rehabilitation, UCL Institute of Neurology, Queen Square, London, UK |
| Emily Todd | Department of Neurodegenerative Disease, Dementia Research Centre, UCL Queen Square Institute of Neurology, London, UK |
| Hanya Benotmane | UK Dementia Research Institute at University College London, UCL Queen Square Institute of Neurology, London, UK |
| Jennifer Nicholas | Department of Medical Statistics, London School of Hygiene and Tropical Medicine, London, UK |
| Kiran Samra | Department of Neurodegenerative Disease, Dementia Research Centre, UCL Queen Square Institute of Neurology, London, UK |
| Martina Bocchetta | Department of Neurodegenerative Disease, Dementia Research Centre, UCL Queen Square Institute of Neurology, London, UK |
| Rachelle Shafei | Department of Neurodegenerative Disease, Dementia Research Centre, UCL Queen Square Institute of Neurology, London, UK |
| Carolyn Timberlake | Department of Clinical Neurosciences, University of Cambridge, Cambridge, UK |
| Thomas Cope | Department of Clinical Neuroscience, University of Cambridge, Cambridge, UK |
| Timothy Rittman | Department of Clinical Neurosciences, University of Cambridge, Cambridge, UK |
| Alberto Benussi | Centre for Neurodegenerative Disorders, Department of Clinical and Experimental Sciences, University of Brescia, Brescia, Italy |
| Enrico Premi | Stroke Unit, ASST Brescia Hospital, Brescia, Italy |
| Roberto Gasparotti | Neuroradiology Unit, University of Brescia, Brescia, Italy |
| Silvana Archetti | Biotechnology Laboratory, Department of Diagnostics, ASST Brescia Hospital, Brescia, Italy |
| Stefano Gazzina | Neurology, ASST Brescia Hospital, Brescia, Italy |
| Valentina Cantoni | Centre for Neurodegenerative Disorders, Department of Clinical and Experimental Sciences, University of Brescia, Brescia, Italy |
| Andrea Arighi | Fondazione IRCCS Ca’ Granda Ospedale Maggiore Policlinico, Neurodegenerative Diseases Unit, Milan, Italy; University of Milan, Centro Dino Ferrari, Milan, Italy |
| Chiara Fenoglio | Fondazione IRCCS Ca’ Granda Ospedale Maggiore Policlinico, Neurodegenerative Diseases Unit, Milan, Italy; University of Milan, Centro Dino Ferrari, Milan, Italy |
| Elio Scarpini | Fondazione IRCCS Ca’ Granda Ospedale Maggiore Policlinico, Neurodegenerative Diseases Unit, Milan, Italy; University of Milan, Centro Dino Ferrari, Milan, Italy |
| Giorgio Fumagalli | Fondazione IRCCS Ca’ Granda Ospedale Maggiore Policlinico, Neurodegenerative Diseases Unit, Milan, Italy; University of Milan, Centro Dino Ferrari, Milan, Italy |
| Vittoria Borracci | Fondazione IRCCS Istituto Neurologico Carlo Besta, Milano, Italy |
| Giacomina Rossi | Fondazione IRCCS Istituto Neurologico Carlo Besta, Milano, Italy |
| Giorgio Giaccone | Fondazione IRCCS Istituto Neurologico Carlo Besta, Milano, Italy |
| Giuseppe Di Fede | Fondazione IRCCS Istituto Neurologico Carlo Besta, Milano, Italy |
| Paola Caroppo | Fondazione IRCCS Istituto Neurologico Carlo Besta, Milano, Italy |
| Pietro Tiraboschi | Fondazione IRCCS Istituto Neurologico Carlo Besta, Milano, Italy |
| Sara Prioni | Fondazione IRCCS Istituto Neurologico Carlo Besta, Milano, Italy |
| Veronica Redaelli | Fondazione IRCCS Istituto Neurologico Carlo Besta, Milano, Italy |
| David Tang-Wai | The University Health Network, Krembil Research Institute, Toronto, Canada |
| Ekaterina Rogaeva | Tanz Centre for Research in Neurodegenerative Diseases, University of Toronto, Toronto, Canada |
| Miguel Castelo-Branco | Faculty of Medicine, University of Coimbra, Coimbra, Portugal |
| Morris Freedman | Baycrest Health Sciences, Rotman Research Institute, University of Toronto, Toronto, Canada |
| Ron Keren | The University Health Network, Toronto Rehabilitation Institute, Toronto, Canada |
| Sandra Black | Sunnybrook Health Sciences Centre, Sunnybrook Research Institute, University of Toronto, Toronto, Canada |
| Sara Mitchell | Sunnybrook Health Sciences Centre, Sunnybrook Research Institute, University of Toronto, Toronto, Canada |
| Christen Shoesmith | Department of Clinical Neurological Sciences, University of Western Ontario, London, Ontario, Canada |
| Robart Bartha | Department of Medical Biophysics, The University of Western Ontario, London, Ontario, Canada; Centre for Functional and Metabolic Mapping, Robarts Research Institute, The University of Western Ontario, London, Ontario, Canada |
| Rosa Rademakers | Center for Molecular Neurology, University of Antwerp |
| Jackie Poos | Department of Neurology, Erasmus Medical Center, Rotterdam, Netherlands |
| Janne M. Papma | Department of Neurology, Erasmus Medical Center, Rotterdam, Netherlands |
| Lucia Giannini | Department of Neurology, Erasmus Medical Center, Rotterdam, Netherlands |
| Rick van Minkelen | Department of Clinical Genetics, Erasmus Medical Center, Rotterdam, Netherlands |
| Yolande Pijnenburg | Amsterdam University Medical Centre, Amsterdam VUmc, Amsterdam, Netherlands |
| Benedetta Nacmias | Department of Neuroscience, Psychology, Drug Research and Child Health, University of Florence, Florence, Italy |
| Camilla Ferrari | Department of Neuroscience, Psychology, Drug Research and Child Health, University of Florence, Florence, Italy |
| Cristina Polito | Department of Biomedical, Experimental and Clinical Sciences “Mario Serio”, Nuclear Medicine Unit, University of Florence, Florence, Italy |
| Gemma Lombardi | Department of Neuroscience, Psychology, Drug Research and Child Health, University of Florence, Florence, Italy |
| Valentina Bessi | Department of Neuroscience, Psychology, Drug Research and Child Health, University of Florence, Florence, Italy |
| Michele Veldsman | Nuffield Department of Clinical Neurosciences, Medical Sciences Division, University of Oxford, Oxford, UK |
| Christin Andersson | Department of Clinical Neuroscience, Karolinska Institutet, Stockholm, Sweden |
| Hakan Thonberg | Center for Alzheimer Research, Division of Neurogeriatrics, Karolinska Institutet, Stockholm, Sweden |
| Linn Öijerstedt | Center for Alzheimer Research, Division of Neurogeriatrics, Department of Neurobiology, Care Sciences and Society, Bioclinicum, Karolinska Institutet, Solna, Sweden; Unit for Hereditary Dementias, Theme Aging, Karolinska University Hospital, Solna, Sweden |
| Vesna Jelic | Division of Clinical Geriatrics, Karolinska Institutet, Stockholm, Sweden |
| Paul Thompson | Division of Neuroscience and Experimental Psychology, Wolfson Molecular Imaging Centre, University of Manchester, Manchester, UK |
| Tobias Langheinrich | Division of Neuroscience and Experimental Psychology, Wolfson Molecular Imaging Centre, University of Manchester, Manchester, UK; Manchester Centre for Clinical Neurosciences, Department of Neurology, Salford Royal NHS Foundation Trust, Manchester, UK |
| Albert Lladó | Alzheimer’s disease and Other Cognitive Disorders Unit, Neurology Service, Hospital Clínic, Barcelona, Spain |
| Anna Antonell | Alzheimer’s disease and Other Cognitive Disorders Unit, Neurology Service, Hospital Clínic, Barcelona, Spain |
| Jaume Olives | Alzheimer’s disease and Other Cognitive Disorders Unit, Neurology Service, Hospital Clínic, Barcelona, Spain |
| Mircea Balasa | Alzheimer’s disease and Other Cognitive Disorders Unit, Neurology Service, Hospital Clínic, Barcelona, Spain |
| Nuria Bargalló | Imaging Diagnostic Center, Hospital Clínic, Barcelona, Spain |
| Sergi Borrego-Ecija | Alzheimer’s disease and Other Cognitive Disorders Unit, Neurology Service, Hospital Clínic, Barcelona, Spain |
| Alexandre de Mendonça | Laboratory of Neurosciences, Institute of Molecular Medicine, Faculty of Medicine, University of Lisbon, Lisbon, Portugal |
| Ana Verdelho | Department of Neurosciences and Mental Health, Centro Hospitalar Lisboa Norte - Hospital de Santa Maria & Faculty of Medicine, University of Lisbon, Lisbon, Portugal |
| Carolina Maruta | Laboratory of Language Research, Centro de Estudos Egas Moniz, Faculty of Medicine, University of Lisbon, Lisbon, Portugal |
| Catarina B. Ferreira | Laboratory of Neurosciences, Faculty of Medicine, University of Lisbon, Lisbon, Portugal |
| Gabriel Miltenberger | Faculty of Medicine, University of Lisbon, Lisbon, Portugal |
| Frederico Simões do Couto | Faculdade de Medicina, Universidade Católica Portuguesa |
| Alazne Gabilondo | Cognitive Disorders Unit, Department of Neurology, Donostia University Hospital, San Sebastian, Gipuzkoa, Spain; Neuroscience Area, Biodonostia Health Research Insitute, San Sebastian, Gipuzkoa, Spain |
| Ana Gorostidi | Neuroscience Area, Biodonostia Health Research Insitute, San Sebastian, Gipuzkoa, Spain |
| Jorge Villanua | OSATEK, University of Donostia, San Sebastian, Gipuzkoa, Spain |
| Marta Cañada | CITA Alzheimer, San Sebastian, Gipuzkoa, Spain |
| Mikel Tainta | Neuroscience Area, Biodonostia Health Research Insitute, San Sebastian, Gipuzkoa, Spain |
| Miren Zulaica | Neuroscience Area, Biodonostia Health Research Insitute, San Sebastian, Gipuzkoa, Spain |
| Myriam Barandiaran | Cognitive Disorders Unit, Department of Neurology, Donostia University Hospital, San Sebastian, Gipuzkoa, Spain; Neuroscience Area, Biodonostia Health Research Insitute, San Sebastian, Gipuzkoa, Spain |
| Patricia Alves | Neuroscience Area, Biodonostia Health Research Insitute, San Sebastian, Gipuzkoa, Spain; Department of Educational Psychology and Psychobiology, Faculty of Education, International University of La Rioja, Logroño, Spain |
| Benjamin Bender | Department of Diagnostic and Interventional Neuroradiology, University of Tübingen, Tübingen, Germany |
| Carlo Wilke | Department of Neurodegenerative Diseases, Hertie-Institute for Clinical Brain Research and Center of Neurology, University of Tübingen, Tübingen, Germany; Center for Neurodegenerative Diseases (DZNE), Tübingen, Germany |
| Lisa Graf | Department of Neurodegenerative Diseases, Hertie-Institute for Clinical Brain Research and Center of Neurology, University of Tübingen, Tübingen, Germany |
| Annick Vogels | Department of Human Genetics, KU Leuven, Leuven, Belgium |
| Mathieu Vandenbulcke | Geriatric Psychiatry Service, University Hospitals Leuven, Belgium; Neuropsychiatry, Department of Neurosciences, KU Leuven, Leuven, Belgium |
| Philip Van Damme | Neurology Service, University Hospitals Leuven, Belgium; Laboratory for Neurobiology, VIB-KU Leuven Centre for Brain Research, Leuven, Belgium |
| Rose Bruffaerts | Department of Biomedical Sciences, University of Antwerp, Antwerp, Belgium; Biomedical Research Institute, Hasselt University, 3500 Hasselt, Belgium |
| Koen Poesen | Laboratory for Molecular Neurobiomarker Research, KU Leuven, Leuven, Belgium |
| Pedro Rosa-Neto | Translational Neuroimaging Laboratory, McGill Centre for Studies in Aging, McGill University, Montreal, Québec, Canada |
| Serge Gauthier | Alzheimer Disease Research Unit, McGill Centre for Studies in Aging, Department of Neurology & Neurosurgery, McGill University, Montreal, Québec, Canada |
| Agnès Camuzat | Sorbonne Université, Paris Brain Institute – Institut du Cerveau – ICM, Inserm U1127, CNRS UMR 7225, AP-HP - Hôpital Pitié-Salpêtrière, Paris, France |
| Alexis Brice | Sorbonne Université, Paris Brain Institute – Institut du Cerveau – ICM, Inserm U1127, CNRS UMR 7225, AP-HP - Hôpital Pitié-Salpêtrière, Paris, France; Reference Network for Rare Neurological Diseases (ERN-RND) |
| Anne Bertrand | Sorbonne Université, Paris Brain Institute – Institut du Cerveau – ICM, Inserm U1127, CNRS UMR 7225, AP-HP - Hôpital Pitié-Salpêtrière, Paris, France; Inria, Aramis project-team, F-75013, Paris, France; Centre pour l'Acquisition et le Traitement des Images, Institut du Cerveau et la Moelle, Paris, France |
| Aurélie Funkiewiez | Centre de référence des démences rares ou précoces, IM2A, Département de Neurologie, AP-HP - Hôpital Pitié-Salpêtrière, Paris, France; Sorbonne Université, Paris Brain Institute – Institut du Cerveau – ICM, Inserm U1127, CNRS UMR 7225, AP-HP - Hôpital Pitié-Salpêtrière, Paris, France |
| Daisy Rinaldi | Centre de référence des démences rares ou précoces, IM2A, Département de Neurologie, AP-HP - Hôpital Pitié-Salpêtrière, Paris, France; Sorbonne Université, Paris Brain Institute – Institut du Cerveau – ICM, Inserm U1127, CNRS UMR 7225, AP-HP - Hôpital Pitié-Salpêtrière, Paris, France; Département de Neurologie, AP-HP - Hôpital Pitié-Salpêtrière, Paris, France |
| Dario Saracino | Sorbonne Université, Paris Brain Institute – Institut du Cerveau – ICM, Inserm U1127, CNRS UMR 7225, AP-HP - Hôpital Pitié-Salpêtrière, Paris, France; Inria, Aramis project-team, F-75013, Paris, France; Centre de référence des démences rares ou précoces, IM2A, Département de Neurologie, AP-HP - Hôpital Pitié-Salpêtrière, Paris, France |
| Olivier Colliot | Sorbonne Université, Paris Brain Institute – Institut du Cerveau – ICM, Inserm U1127, CNRS UMR 7225, AP-HP - Hôpital Pitié-Salpêtrière, Paris, France; Inria, Aramis project-team, F-75013, Paris, France; Centre pour l'Acquisition et le Traitement des Images, Institut du Cerveau et la Moelle, Paris, France |
| Sabrina Sayah | Sorbonne Université, Paris Brain Institute – Institut du Cerveau – ICM, Inserm U1127, CNRS UMR 7225, AP-HP - Hôpital Pitié-Salpêtrière, Paris, France |
| Catharina Prix | Neurologische Klinik, Ludwig-Maximilians-Universität München, Munich, Germany |
| Elisabeth Wlasich | Neurologische Klinik, Ludwig-Maximilians-Universität München, Munich, Germany |
| Olivia Wagemann | Neurologische Klinik, Ludwig-Maximilians-Universität München, Munich, Germany |
| Sandra Loosli | Neurologische Klinik, Ludwig-Maximilians-Universität München, Munich, Germany |
| Sonja Schönecker | Neurologische Klinik, Ludwig-Maximilians-Universität München, Munich, Germany |
| Tobias Hoegen | Neurologische Klinik, Ludwig-Maximilians-Universität München, Munich, Germany |
| Jolina Lombardi | Department of Neurology, University of Ulm, Ulm |
| Sarah Anderl-Straub | Department of Neurology, University of Ulm, Ulm, Germany |
| Adeline Rollin | CHU, CNR-MAJ, Labex Distalz, LiCEND Lille, France |
| Gregory Kuchcinski | Univ Lille, France; Inserm 1172, Lille, France; CHU, CNR-MAJ, Labex Distalz, LiCEND Lille, France |
| Maxime Bertoux | Inserm 1172, Lille, France; CHU, CNR-MAJ, Labex Distalz, LiCEND Lille, France |
| Thibaud Lebouvier | Univ Lille, France; Inserm 1172, Lille, France; CHU, CNR-MAJ, Labex Distalz, LiCEND Lille, France |
| Vincent Deramecourt | Univ Lille, France; Inserm 1172, Lille, France; CHU, CNR-MAJ, Labex Distalz, LiCEND Lille, France |
| Beatriz Santiago | Neurology Department, Centro Hospitalar e Universitario de Coimbra, Coimbra, Portugal |
| Diana Duro | Faculty of Medicine, University of Coimbra, Coimbra, Portugal |
| Maria João Leitão | Centre of Neurosciences and Cell Biology, Universidade de Coimbra, Coimbra, Portugal |
| Maria Rosario Almeida | Faculty of Medicine, University of Coimbra, Coimbra, Portugal |
| Miguel Tábuas-Pereira | Neurology Department, Centro Hospitalar e Universitario de Coimbra, Coimbra, Portugal |
| Sónia Afonso | Instituto Ciencias Nucleares Aplicadas a Saude, Universidade de Coimbra, Coimbra, Portugal |
