# Supplementary material for: Differential impairment of cerebrospinal fluid synaptic biomarkers in the genetic forms of frontotemporal dementia
Source: Alzheimers Res Ther. 2022 Aug 31;14:118. doi: 10.1186/s13195-022-01042-3 (PMC9429339; doi:10.1186/s13195-022-01042-3)
Supplement: Supplementary file 3 — Additional file 3: Appendix 2. IS preparation and LC-MS settings for theanalysis of the synaptic protein panel. [file 13195_2022_1042_MOESM3_ESM.docx]

**Appendix 2**

**IS preparation and LC-MS settings for the analysis of the synaptic protein panel**

Stable-isotope-labeled peptides (JPT Peptide Technologies, Berlin, Germany; SpikeTides) and internal standards (IS), were mixed equally to a final concentration of 32 fmol/µL, and 25 µL of the IS mixture was added to 100 µL CSF samples. Reduction was performed by the addition of 25 µL of 30 mM 1,4-dithiothreitol (5 mM final concentration, Sigma-Aldrich) and incubation at 60 °C for 30 minutes. This was followed by alkylation by the addition of 25 µL of 70 mM iodoacetamide (10 mM final concentration, Sigma-Aldrich) and incubation for 30 minutes at room temperature. Finally, digestion was executed by the addition of 25 µL of 0.02 µg/µL trypsin/Lys-C mix (Mass spectrometry grade, Promega Co, Madison, WI, USA) and incubation at 37 °C for 18 hours. Lastly, desalting of the samples were performed by solid-phase extraction using Oasis 30 µm HLB 96-well µElution Plates (Waters Co., Milford, MA, USA). The samples were dried in a vacuum centrifuge before storage at −80 °C pending analysis and reconstituted in 100 µL of 50 mM NH_4_HCO_3_ for analysis.

|  | **Parameter** | **Setting** |
| --- | --- | --- |
| **LC** | Sample injection volume | 40 µL |
|  | Flow-rate | 0.3 mL/min |
|  | Gradient | Broken; 5–20%B (20 min), 20–35%B (7 min) |
|  | Total cycle time | 30 min |
|  | Mobile phase A | 0.1% formic acid in water (v/v) |
|  | Mobile phase B | 0.1% formic acid/84% acetonitrile in water (v/v) |
| **Electrospray** | Mode | Positive |
|  | Gas temperature | 220 °C |
|  | Gas flow | 15 L/min |
|  | Nebulizer pressure | 40 psi |
|  | Sheath gas temperature | 200 °C |
|  | Sheath gas flow | 11 L/min |
|  | Capillary voltage | 3500 V |
|  | Nozzle voltage | 500 V |
| **iFunnel** | Mode | Positive |
|  | High-pressure radio frequency | 200 V |
|  | Low-pressure radio frequency | 160 V |
|  |  |  |
| **MRM method** | Retention time window | 0.8 min |
|  | Collision energies | Individually optimized per transition |
|  | Cell accelerator voltage | Individually optimized per transition |
